# The Story of a Hitchhiker: Population Genetic Patterns in the Invasive Barnacle *Balanus*(*Amphibalanus*) *improvisus* Darwin 1854

**DOI:** 10.1371/journal.pone.0147082

**Published:** 2016-01-28

**Authors:** Anna-Lisa Wrange, Gregory Charrier, Anne Thonig, Magnus Alm Rosenblad, Anders Blomberg, Jonathan N. Havenhand, Per R. Jonsson, Carl André

**Affiliations:** 1 University of Gothenburg, Department of Marine Sciences—Tjärnö, Sweden; 2 Institut Universitaire Européen de la Mer (IUEM), Technopôle Brest-Iroise, Plouzané, France; 3 Roskilde University, Department of Environmental, Social and Spatial Change, Roskilde, Denmark; 4 University of Gothenburg, Department of Chemistry and Molecular Biology, Gothenburg, Sweden; Kunming Institute of Zoology, Chinese Academy of Sciences, CHINA

## Abstract

Understanding the ecological and evolutionary forces that determine the genetic structure and spread of invasive species is a key component of invasion biology. The bay barnacle, *Balanus improvisus* (= *Amphibalanus improvisus*), is one of the most successful aquatic invaders worldwide, and is characterised by broad environmental tolerance. Although the species can spread through natural larval dispersal, human-mediated transport through (primarily) shipping has almost certainly contributed to the current global distribution of this species. Despite its worldwide distribution, little is known about the phylogeography of this species. Here, we characterize the population genetic structure and model dispersal dynamics of the barnacle *B*. *improvisus*, and describe how human-mediated spreading via shipping as well as natural larval dispersal may have contributed to observed genetic variation. We used both mitochondrial DNA (cytochrome c oxidase subunit I: COI) and nuclear microsatellites to characterize the genetic structure in 14 populations of *B*. *improvisus* on a global and regional scale (Baltic Sea). Genetic diversity was high in most populations, and many haplotypes were shared among populations on a global scale, indicating that long-distance dispersal (presumably through shipping and other anthropogenic activities) has played an important role in shaping the population genetic structure of this cosmopolitan species. We could not clearly confirm prior claims that *B*. *improvisus* originates from the western margins of the Atlantic coasts; although there were indications that Argentina could be part of a native region. In addition to dispersal via shipping, we show that natural larval dispersal may play an important role for further colonisation following initial introduction.

## Introduction

The number of non-native species being introduced to coastal and estuarine habitats has increased dramatically during the past century; largely due to anthropogenic activities including increased shipping intensity and aquaculture trading [1,2]. In many cases, these introductions have resulted in major disturbances of native ecosystems as well as economic losses, and biological invasions are today considered a major contributor to global environmental change and a threat to biodiversity [3–5]. Knowledge of the genetic structure and dispersal dynamics of non-indigenous species is important not only for management of invasive species, but also for improving our understanding of ecological and evolutionary processes related to colonisation of new environments, e.g. the role of genetic diversity, life history traits, environmental tolerance and gene flow via larval dispersal, for successful invasions [6–9].

Dispersal dynamics in the marine realm are generally complex and not well understood [10]. Dispersal of free-drifting larvae depends not only on oceanographic conditions, but also on life history characteristics of the individual species, including larval form, swimming ability, nutritional modes and reproductive timing [11,12]. In general, species with the capacity to disperse over long distances are traditionally expected to have weak population structure since a few migrants may be sufficient to homogenise local genetic variation [13,14]. However, multiple studies have shown surprisingly large genetic differentiation over small geographical scales in a number of marine species with pelagic larval dispersal [15–20]. Genetic differentiation between populations despite high gene flow can e.g. be the result of non-random settlement or selection through post-settlement mortality [21–23]. In addition to natural larval dispersal, human-mediated translocation often increases the complexity of the observed genetic patterns [24–26].

Genetic markers are typically used in tracing origins of populations as well as understanding population structure and dispersal mechanisms [25,27–29]. Sequence-based mitochondrial DNA (mtDNA) has commonly been used to infer demographical history, e.g. founder events and demographic expansions associated with post-glacial colonisation (e.g. [30,31]). Frequency-based markers such as microsatellites and AFLPs, on the other hand, are often used to detect more recent and fine-scale differentiation between populations [32–34] and for studying how ecological factors influence contemporary partitioning of genetic variation [35]. However, genetic structure does not always mirror gene flow, since many populations are not in migration-drift equilibrium [16,36]. Genetic structure is the result of contemporary levels of gene flow superimposed on the traces of phylogeographical and demographical history. Nevertheless, genetic tools can provide relevant baseline information about population structure and potential for local adaptations to evolve. Furthermore, combining genetic data with oceanographic modelling has provided new interesting ways to detect and understand gene flow between populations [37–40].

The acorn barnacle *Balanus improvisus* (= *Amphibalanus improvisus*) has invaded shallow coastal environments worldwide and is particularly common in estuaries and low salinity environments where it often forms dense populations [41–43]. There are several factors contributing to the successful introduction and spread of this species. First, *B*. *improvisus* is a major fouling organism, attaching to hard surfaces such as ship hulls, thus being transported efficiently over large distances [44–46]. Second, the species has a broad tolerance to many abiotic factors, including salinity [47,48], temperature and pH [49]. Broad environmental tolerance is a common trait for many successful invaders [50]. Finally, as in other barnacles, *B*. *improvisus* produces free-swimming larvae, which remain planktonic for several weeks [51], during which they disperse with oceanographic currents.

Despite being a major fouling organism invading coastal areas worldwide, little is known about the origin, colonisation history and present population genetic structure of this species. *B*. *improvisus* is commonly described as originating from America’s Atlantic coast [41,52], however no clear evidence supporting this statement is available. Charles Darwin, who first described the species, recorded populations from the north- and southeast coasts of America, but also from several localities in Europe, including the UK [53]. *B*. *improvisus* was first recorded along the Pacific coast of the US in 1853, possibly after human introduction via shipping during the gold rush or oyster imports [54]. In the western Pacific, the first recordings of the species from Japan were not made until the 1950’s [55]. Many of the early recordings of *B*. *improvisus* from around the world were made before or around the time that the species was first described by Darwin [54,56,57], leading to confusion about precisely which species were recorded, especially since several other acorn barnacles have similar morphology (e.g. *Balanus eburneus*, *B*. *crenatus*, *B*. *subalbidus*) [53]. The timing of these early observations also makes it difficult to estimate historical colonisation patterns based on first observations in the literature.

Although *B*. *improvisus* is a highly euryhaline species, it performs best in low salinity [48,58] and has invaded several large brackish seas. It was first observed in the Black Sea in 1899 [59] and when the Volga-Don Canal opened in 1952, it penetrated into the Caspian Sea and became locally dominant within three years [60]. In the Baltic Sea, *B*. *improvisus* was first recorded in 1844 from Königsberg (today Kaliningrad)—ten years before the species was officially described by Darwin in 1854 [53,57]. From this putative dispersal centre in the southern Baltic it spread very rapidly and became common, especially in ports [43,56]. There are few records of this species from the Swedish east coast before the 1920’s but by the 1990’s, *B*. *improvisus* reached its northernmost limit of distribution at the Northern Quark, at 63° N [52]. The semi-enclosed Baltic Sea is characterized by a steep salinity gradient and has limited connectivity with the adjacent North Sea/Atlantic, which has resulted in both genetic and phenotypic differentiation in many species–and in some cases local adaptations–in the Baltic environment [61,62]. Despite being a common, microhabitat-forming species in the Baltic Sea [43,52], there is limited knowledge about the dispersal dynamics and genetic structure of *B*. *improvisus* in the Baltic Sea. Furman [63,64] used allozymes to study population differences in the Baltic Sea but found weak differentiation between populations and no clear geographical structuring (see also [61]).

Using mitochondrial and microsatellite DNA markers, we addressed the following questions: i) What role might natural larval dispersal *versus* human-mediated dispersal via shipping play in shaping the population structure? ii) Can the invasive history of *B*. *improvisus* be clarified despite multiple dispersal modes and potentially high gene flow?

## Methods

No specific permits were required for sampling. No specific permissions were required for the locations/activities in this study. The sampling locations are not privately owned or protected in any way. This study did not involve endangered or protected species.

### Sample collection and DNA extraction

A total of 454 individuals of the acorn barnacle *Balanus improvisus* were collected during 2008–2013. The 14 sampling locations were: Coos Bay (Oregon, Pacific coast of USA); Chesapeake Bay (Atlantic USA); North Carolina (Atlantic USA); Mar Chiquita Lagoon (Argentina); Seine estuary in the eastern English Channel (France); Sozopol, Black Sea (Bulgaria); western Caspian Sea (Iran); Tokyo Harbour (Japan) as well as six locations in the Skagerrak-Baltic Sea area; Saltö (Sweden) in the Skagerrak sea, Kiel fjord (Germany), Muuga Bay (Estonia) and Torhamn, Öregrund and Umeå on the Swedish east coast (Table 1, S1 Table). The samples were collected by removing juvenile or adult individuals of *B*. *improvisus* from rocks and other hard surfaces that were present in the intertidal/shallow area at each sample site. No samples were taken from boat hulls. The specimens were preserved in 96% ethanol or on FTA cards (Whatman) and stored at -20°C. The morphology of the barnacles in ethanol was checked under a stereomicroscope to confirm the species, according to [65]. DNA extraction was performed using the Nucleospin Tissue Kit (Macherey Nagel) following either the protocol for tissue or FTA cards. Unless the specimens were very small, only the cirri (legs) of the barnacles were used. The DNA concentration and quality was analysed with a Nanodrop (Thermo Scientific). Except for the populations from the eastern USA (NC and CB), around 30 individuals per locality (N = 24–57) were extracted for the subsequent PCR amplifications (Table 1**)**.

**Table 1:** Sampling information and genetic diversity for mtDNA (COI) and microsatellite markers for the barnacle *B*. *improvisus*.

	Mitochondrial DNA						Microsatellites			
Sampling location	N	# hapl	# Private hapl	Priv. hapl/N	*h*	π	N	He	Ho	Ar
Chesapeake Bay (CB)	7	2	0	0.00	0.286	0.0009	-	-	-	-
North Carolina (NC)	7	3	1	0.14	0.524	0.0012	-	-	-	-
Argentina (AR)	30	17	5	0.17	0.920	0.0063	30	0.785	0.390	8.2
France (FR)	29	19	10	0.34	0.916	0.0055	29	0.807	0.422	9.1
Saltö (SA)	49	27	13	0.27	0.933	0.0061	56	0.787	0.445	9.0
Kiel (KL)	30	18	6	0.20	0.922	0.0045	27	0.756	0.505	8.9
Torhamn (TO)	29	21	7	0.24	0.929	0.0060	33	0.828	0.428	8.6
Estonia (ES)	30	21	8	0.27	0.963	0.0058	30	0.803	0.472	8.1
Öregrund (OR)	57	22	8	0.14	0.751	0.0039	57	0.809	0.455	8.8
Umeå (UM)	50	23	10	0.20	0.851	0.0045	50	0.819	0.408	8.5
Black Sea (BL)	26	9	0	0.00	0.815	0.0025	24	0.820	0.492	7.5
Caspian Sea (CS)	30	15	4	0.13	0.832	0.0040	30	0.806	0.466	7.7
Japan (JP)	32	20	7	0.22	0.950	0.0069	33	0.763	0.400	8.4
Pacific US (PU)	42	24	12	0.29	0.929	0.0062	44	0.798	0.459	8.8
TOTAL	448	139					443			

N = sample size; # hapl = number of haplotypes; # private hapl = number of private haplotypes; *h* = haplotype diversity; π = nucleotide diversity; He = expected heterozygosity; Ho = observed heterozygosity; Ar = allelic richness (based on 17 individuals).

### Genotyping of mitochondrial COI

Primers for the PCR amplification of the mitochondrial cytochrome c oxidase subunit I gene (COI) were designed based on mitochondrial sequences from the *Balanus* sequencing project currently carried out within the Linneaus Centre for Marine Evolutionary Biology at University of Gothenburg (www.cemeb.science.gu.se/research/imago-marine-genome-projects). For the PCR, extracted DNA was either diluted to concentrations of 10 ng/μL or used without dilution when the concentration was below 20 ng/μL. PCR reactions were performed in 50μL volume, containing 5 μL of template, 1X RBC buffer with 1.5 mM MgCl_2_, 1.5μM dNTPs, 0.13 U RBC Taq polymerase (RBC Bioscience) and 0.2 μM each of two of the primers (BiCOI-F-ext: 5'-TCTGAAACTCTTACTTTTGACCG-3', BiCOI-R-ext: 5'-CATTACCTGTTTTAGCTGGTGC-3'; Integrated DNA Technologies). The PCR program consisted of an initial pre-cycle of 3 min at 94°C, 2 min at 55°C and 1 min at 72°C, which was followed by 35 cycles of 30 s at 94°C, 30 s at 55°C and 1 min at 72°C, and finally an extension at 72°C for 7 min. Afterwards, 3.5 μL of the PCR products were visualized on a 1.5% agarose gel stained with GelRed (Invitrogen). This amplification resulted in 850 bp long fragments. However, for the specimens from the eastern USA, Öregrund, and partly Japan and the Black Sea, a nested PCR was necessary using the external primer pair in the first step and an internal primer pair (BiCOI-F int: 5'-TTGATGATACTACATTTCAGGCAG-3', BiCOI-R int: 5'- GATAATCCGAATATCGACGAGG-3') in the second step. This in turn, resulted in a 738 bp long fragment. The PCR products were purified using the Cycle Pure Kit (Omega Biotek), and sequenced by Macrogen Europe, in both directions using the internal primer pair above. The sequences have been deposited in the Dryad Database (https://datadryad.org/) with the accession number DOI: 10.5061/dryad.sf2v0.

### Development and genotyping of microsatellites

Three different development approaches were used to obtain microsatellites for *Balanus improvisus*. First, microsatellites were isolated by the company Genetic Identification Service Inc. (GIS Inc., Chatsworth, USA; http://genetic-id-services.com), which screened four libraries enriched for tetranucleotide (AAAC), (CATC), (TACA), and (TAGA) motifs, following their proprietary protocol. Out of the 69 unique microsatellite-containing clones that were obtained from GIS, primer pairs were successfully designed in 36 clones with a minimum of seven di- or tetranucleotide repeats using PRIMER3 [66] implemented in GENEIOUS PRO v.5.1.7 ([67]; Biomatters Ltd). The primer design was performed according to the following parameters: optimal primer length = 20 bp (range = 18–25 bp), optimal GC content = 50% (range = 30–80%), optimal Tm = 60°C (range = 59–61°C) with a Tm difference not larger than 1°C between the forward and reverse primers from the same pair and product length = 100–400 bp. As a second development approach, microsatellites were identified with Tandem Repeat Finder [68] from an EST (expressed sequence tags) library, containing both Sanger and 454 sequences from *B*. *improvisus* cyprid larvae (unpublished: www.cemeb.science.gu.se/research/imago-marine-genome-projects). Out of 593 microsatellites identified with 2–6 bp motifs, 53 unique di-, tri- and tetranucleotide microsatellites were selected for primer design (>7 repeats, flanking regions >40 bp, perfection >87%), and primers pairs were successfully designed for 36 loci with PRIMER3 implemented in GENEIOUS PRO, following the same parameters as described above. Finally, the third microsatellite development approach involved the identification of microsatellite loci in a *B*. *improvisus* (a single individual from Saltö, Sweden) genomic assembly (500 Mbases) made from two 150 bp and 300 bp libraries containing a total of 83 million paired-end (2x100bp) Illumina reads (unpublished: www.cemeb.science.gu.se/research/imago-marine-genome-projects). In this library, 3916 di-, tri- and tetranuclotide microsatellites with a minimum of seven repeats and flanking regions >100 bp were identified with REPEATMASKER 3.3 [69], and 128 tri- and tetra-nucleotide microsatellites with 16–50 repeats were selected for primer design. Finally, primers were successfully designed for 36 loci with PRIMER3 implemented in GENEIOUS PRO, with the following parameters: optimal primer length = 20 bp (range = 18–25 bp), optimal GC content = 50% (range = 30–80%), optimal Tm = 60°C (range = 58–62°C) with a Tm difference not larger than 2°C between the forward and reverse primers from the same pair and optimal product length = 200 bp (range = 100–300 bp). All the primers were subsequently checked manually. A ‘universal tail’ was added to the 5’-end of each forward primer [70]. Three distinct ‘universal tails’ were used (tail1, tail2 or tail4), as described by Real et al. [71]. A PIG-tail (50-GTTTCTT) was also added to the 5’-end of each reverse primer to avoid ‘plus-A’ PCR artefacts [72]. Some microsatellites were compared with and without tails, with no difference found in amplification success between the two methods. Microsatellite amplifications were carried out in 10-μL reaction volumes, containing 1 μL of template DNA (10–30 ng/μL), 1X PCR buffer (Mg^2+^ free), 0.2 mM of each dNTP, 1.5–2.5 mM MgCl_2_ (S2 Table), 0.025 U of RBC Taq polymerase (RBC Bioscience), 0.125 μM of reverse primer, 0.0125 μM of tailed forward primer (Integrated DNA Technologies), 0.125 μM of “universal” primer end labelled with WellRED dye (D2, D3 or D4, Sigma-Aldrich). A touchdown procedure was incorporated in the thermal cycling regime to increase the stringency of the PCRs. The PCR cycling conditions were as follows (alternative step lengths in *italic*): 94°C for 3 min, Ta1 for 2 min, 72°C for 1 min; (94°C for 30 s, Ta1–1 C for 30(*40*) s [-1°C per cycle until Ta2], 72°C for 1 min) x 11 cycles; (94°C for 30 s, Ta2 for 30(*40*) s, 72°C for 1 min) x 23 cycles; 72°C for 5(*7/10*) min. Details of the PCR protocol, i.e. MgCl_2_ concentration, number of PCR cycles and type of ‘universal’ tail, are provided in S2 Table. Sizing of PCR products was performed on a Beckman Coulter’s CEQ 8000 automated sequencer where all lanes included a 400-bp ladder. Allele sizes were scored with the software CEQ 8000 Genetic Analysis System (version 8.0.52). In spite of multiple PCR adjustments and primer re-designs, only four microsatellites out of the 108 loci tested were successfully amplified, produced consistent and clearly defined bands and displayed polymorphism: two loci from the GIS library (tetranucleotides B4 and C103), one locus from the EST library (trinucleotide E29), and one from the genomic library (tetranucleotide E42). Genotype lists for these four microsatellite loci have been deposited in the Dryad Database (https://datadryad.org/) with the accession number DOI: 10.5061/dryad.sf2v0).

### Analysis of COI sequences

The COI sequences were edited and aligned using GENEIOUS PRO 6.1.6 (http://www.geneious.com, [73]). Primers and low quality sequence ends were trimmed, and all polymorphic sites were checked by eye on the chromatograms. Thereafter, each forward sequence was aligned with its respective reverse sequence to generate a consensus sequence of 694 bp for every individual. The resulting consensus sequences were aligned again and subsequently used for further analysis.

Molecular diversity indices including haplotype diversity (*h*, [74]), nucleotide diversity (*π*; [74]), number of polymorphic sites and haplotypes were obtained using ARLEQUIN v.3.5 [75]. The appropriate nucleotide substitution model and the gamma distribution shape parameter for rate heterogeneity among sites were assessed with jMODELTEST2 [76,77]. Results obtained from JMODELTEST2 showed that the best substitution model among those proposed in ARLEQUIN was the Tamura and Nei model with a gamma correction (α = 0.023) for heterogeneity of mutation rates (4^th^ best model choice according to AIC and BIC criteria).

Genealogical relationships among COI haplotypes were examined by reconstructing a haplotype network following the parsimony method of Templeton et al. [78] implemented in TCS v. 1.21 [79]. However, this network was very unclear since it displayed numerous multiple connections (loops). Therefore, a minimum spanning tree was built in HAPSTAR [80] to indicate the most likely connections among haplotypes. Thereby all the loops that were observed in TCS were resolved by selecting the most likely connection and deleting other alternative connections, thus converting the parsimony network into a minimum spanning tree. In addition, the phylogenetic relationships of haplotypes were explored with a maximum likelihood phylogenetic tree that was built with PHYML [77]. The HKY substitution model [81] with a gamma correction α = 1.015 and a proportion of invariant sites p-inv. = 0.691 was selected as the optimal model among those available in PHYML, according to JMODELTEST2. The robustness of branches was tested with 100 bootstraps, and two COI sequences of *Balanus eburneus* were used as an out-group for this maximum likelihood analysis.

We estimated pair-wise genetic differentiation between populations using the fixation index Φ_ST_ [82] obtained from ARLEQUIN, which includes information on both haplotype frequencies as well as information on molecular distance (using the Tamura-Nei model with gamma correction α = 0.023). Significance was tested with 10,000 permutations and False Discovery Rate (FDR) was calculated to take into account multiple comparisons [83]. Pairwise Φ_ST_ were further linearized following [84] as implemented in ARLEQUIN v.3.5.1.2 and the paired genetic distances between populations (matrix) were visualized using non-metric multidimensional scaling (MDS) performed with metaMDS in the vegan package in R (R Development Core Team, 2006; http://www.R-project.org). A hierarchical analysis of molecular variance (AMOVA) [82] using the Tamura-Nei model with gamma correction (α = 0.023) was performed using ARLEQUIN to further explore the distribution of the genetic variability (in mtDNA) between groupings in the Baltic Sea that were identified in the MDS. The significance of the observed variances for each hierarchical comparison was tested by 10,000 permutations. The historical demography of *B*. *improvisus* was investigated using a mismatch distribution analysis [85,86]. The Harpending’s raggedness (R) test [87] was used to determine if the observed mismatch distribution was taken from an expanding or stationary population. Tajima’s *D* [88] and Fu’s *F*_S_ [89] that test for departure from population equilibrium were calculated using ARLEQUIN, and significance was evaluated based on 10,000 simulations. Significance level for Fu’s *F*_S_ test was set to 0.02 according to recommendation in the ARLEQUIN 3.5 manual.

### Analysis of microsatellite data

The software MICRO-CHECKER [90] was used to investigate the presence of null alleles, stutter and large allele dropout. Since potential null alleles were identified in three out of four loci (B4, E24, E42) the software FreeNA [91] was used to estimate and correct for null allele frequencies applying the ENA correction method. GENEPOP v. 4.2 [92] was used to calculate number of alleles (*N*_A_), observed (*H*_O_) and expected (*H*_E_) heterozygosity, and to test for deviations from Hardy–Weinberg equilibrium and linkage disequilibrium. *F*_IS_ was estimated and tested using the probability tests within GENEPOP. Microsatellite allelic richness (Ar) was calculated using the rarefaction method in FSTAT [93]. Population differentiation among samples was estimated using the *F*_ST_ estimator *θ* [94], and in FreeNA using ENA correction for null alleles. Statistical significance was calculated for uncorrected data using permutation tests implemented in FSTAT, and False Discovery Rate (FDR) was used to account for multiple comparisons [83]. For null allele corrected data, statistical significance could not be estimated since data contained less than five loci [91]. The BOTTLENECK software (version 1.2.02; [95]) was applied to investigate population declines, using the TPM model with 70–90% stepwise mutations, a variance of 10–30, and based on 10000 interactions. The Wilcoxon one-tailed test was used to check for significant heterozygosity excess.

### Inference of introduction history using ABC modeling

The introduction history of *B*. *improvisus* was explored with approximate Bayesian computation analyses (ABC). ABC analyses of both the four microsatellite markers and mitochondrial sequences of the COI locus were analysed with the program DIYABC v2.1.0 (www1.montpellier.inra.fr/CBGP/diyabc/), described in [96, 97]. We tested four global scenarios of sequential introductions where we contrasted likely scenarios based on known and assumed introductions with possible alternative scenarios. Finally, we tested two more local scenarios for the invasion of *B*. *improvisus* in the Baltic Sea. The tested scenarios are shown in S1 Fig. Briefly, scenario 1 represents an introductory history where the east coast of the Americas (here represented by Argentina) is assumed to be the native region for *B*. *improvisus* and with one introductory branch to the Pacific coast of America, Japan, the Black Sea and the Caspian Sea, and another introductory branch to Europe (France) and later into the Baltic Sea (Kiel, Torhamn, Estonia, Öregrund and Umeå samples). Scenario 2 instead, assumes the west coast of the Americas to be the native region with one introductory branch to Japan, the Black Sea and the Caspian Sea, and another sequence to the east coast of the Americas then to Europe and into the Baltic Sea. Scenario 3 assumes an early introduction to Europe and subsequent dispersal into the Baltic Sea and later to the Black and the Caspian Seas. Finally, Scenario 4 assumes that *B*. *improvisus* originates from Europe. We also simulated two scenarios for the invasion into the Baltic Sea (S1E and S1F Fig). Scenario 1 assumes that the Baltic Sea was colonized from the south to the north through the North Sea, while scenario 2 assumes that the northern part of the Baltic Sea has a Ponto-Caspian origin, as is the case for some introduced species in the Baltic Sea [52]. We here grouped the Kiel, Torhamn and Estonia samples into the group “Baltic Sea south” (BA-S) and the Öregrund and Umeå samples into “Baltic Sea north” (BA-N).

Since there is little information about present and historical demographic parameters, we sampled priors from a wide range of uniform distributions. Effective population sizes for all populations ranged between 10,000 and 100,000 for simulations of COI and between 10,000 and 200,000 for simulations of microsatellites. For the COI we used the mutation model by Tamura-Nei and sampled from a uniform distribution of mean mutation rates between 10^−8^ and 10^−6^. For the microsatellite loci the Generalized Stepwise Mutation model was used [98] with a uniform distribution of mean mutation rates between 10^−4^ and 10^−3^. Times of new introductions (population splits) ranged between 10–10,000 generations for all introductions. The genetic variation within and between populations was calculated using a set of summary statistics. For microsatellites we used mean number of alleles and expected heterozygosity within populations, and the pairwise comparisons *F*_*ST*_ and *dμ*^*2*^ between populations. For the COI sequences we used mean number of haplotypes and mean of pairwise differences within populations, and *F*_*ST*_ and mean number of pairwise differences between populations. We simulated between 10^6^ to 4∙10^6^ data sets per scenario. The distributions of summary statistics based on the sampled priors were reasonably close to observed data (S2 Fig). The posterior probabilities of the tested scenarios was estimated with a polychotomic weighted logistic regression on the 1% of simulated data sets most similar to the observed data with the proportion of the target scenario as the dependent variable and the differences between observed and simulated data set summary statistics as the independent variables.

### Correlating genetic structure to geographic distance and oceanographic connectivity

To test the hypothesis of isolation-by-distance, correlation between pairwise linearized Φ_ST_ for COI and *F*_ST_ (with and without null allele correction) for microsatellites [84], versus geographical distances between locations was analysed using a Mantel test [99] implemented in R with the function mantel.test in the ncf package (tested with 1000 permutations). Geographical distances were based on the shortest shipping routes between the closest major ports to the sampling sites (calculated at: www.sea-distances.com) and distances obtained in nautical miles were converted into km.

We also tested if genetic differentiation between populations was correlated with oceanographic connectivity in the Baltic Sea. The connectivity between sites was estimated using a biophysical model, where velocity fields from an ocean circulation model were combined with a Lagrangian particle-tracking routine to simulate drift trajectories representing the dispersal of barnacle larvae. Velocity fields were first produced with the BaltiX model, which is a regional Baltic-North Sea configuration of the NEMO ocean model [100]. The spatial resolution is 3.7 km in the horizontal and 56 depth intervals in the vertical ranging from 3 to 22 m. The model has a free surface and allows the grid boxes to stretch and shrink vertically to accurately model the tides without generating empty grid cells at low tide. For a more elaborate description and some validation results see [101]. Dispersal of virtual larvae was simulated with the Lagrangian model TRACMASS [102], which produced multiple dispersal trajectories using the velocity field data from the BaltiX circulation model. Velocity fields were updated for all grid boxes in the model domain every three hours while the trajectory calculations were done with a 15-minute time step. To mimic expected larval behaviour of barnacle larvae we only allowed dispersal in the top 12 m [51]. We further assumed that spawning occurred from June to August [103–105] and that the pelagic larval duration lasted between 20 and 30 days [106]. The dispersal modelling was repeated for eight years (1995–2002) to account for short-term climate variations due to the North-Atlantic Oscillation cycle [107]. Within each locality consisting of 10 model grid cells (3.7 x 3.7 km^2^) we released a total of 47040 particles. Dispersal probabilities between the seven localities were calculated by summing all the trajectories starting in locality *i* having end positions within locality *j* normalized by the total number of simulated trajectories from locality *i*. Also the probability to disperse within any locality was calculated. In addition, we calculated the multi-generation connectivity where stepping-stone dispersal was allowed over 16 and 32 single-generation dispersal events, and summed over all possible dispersal routes [108]. We chose to include a broad range of habitats since the distribution of *B*. *improvisus* is poorly described in the Baltic Sea, and to reduce the risk of underestimating connectivity. However, we excluded all areas below 100 m, which are anoxic. Tests for correlations between multi-generational connectivity and linearized genetic differentiation (*F*_ST_ /1- *F*_ST_) were performed using the same Mantel test as described above. The data on linearized *F*_ST_ for genetic differentiation are symmetric between localities. This is generally not the case for oceanographic connectivity and in the study area there are marked asymmetries in water transport. To allow for a Mantel test we made the connectivity matrices symmetric by calculating the minimum connectivity between each pair of localities, which may arguably be best correlated with increasing *F*_ST_. Minimum connectivity also explained more of the genetic differentiation compared to the maximum, as well as to the arithmetic and geometric means of connectivity.

## Results

### COI sequence variation and diversity within populations

**Fig 1:**
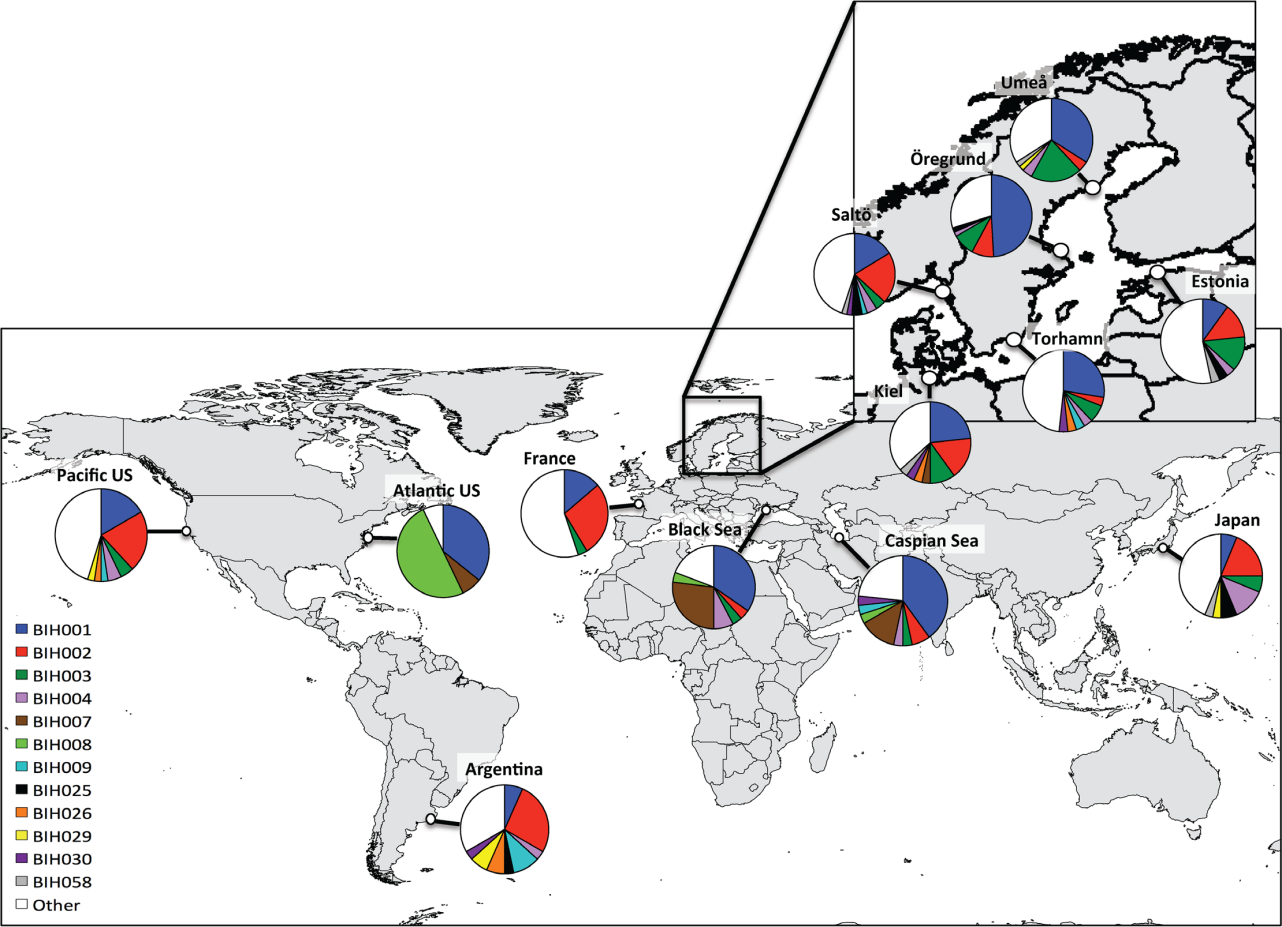
Distribution of haplotypes in the different populations of *B*. *improvisus*. The twelve most common haplotypes (represented by five or more individuals) are colour-coded and the “white proportion” contains all the haplotypes represented by five or less individuals including unique haplotypes.

**Fig 2:**
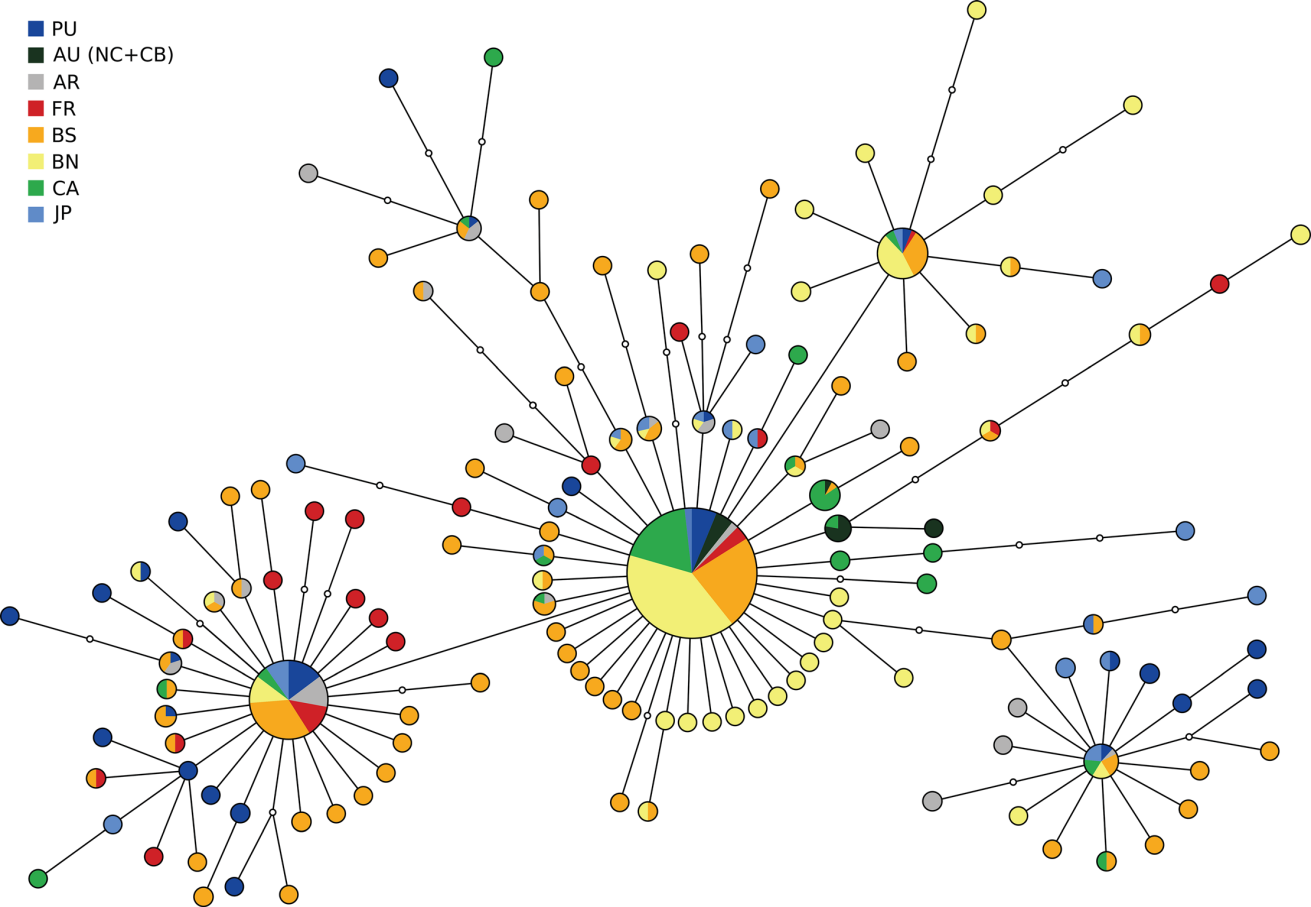
A minimum spanning haplotype network based on a 694 bp fragment of the mtDNA COI gene in *B*. *improvisus*. Each node represents one mutational step and the size of each circle represents the frequency of a haplotype. The pie chart colours represent different geographical groupings of populations; PU = Pacific US, AU = Atlantic US (North Carolina and Chesapeake Bay combined), AR = Argentina, FR = France, BS = Baltic South (including Kiel, Torhamn, Estonia and Saltö), BN = Baltic North (Öregrund and Umeå), CA = Central Asia (Caspian Sea and Black Sea) and JP = Japan. Small empty circles (nodes) represent non-sampled haplotypes.

A 694 bp sequence of the cytochrome oxidase I (COI) was successfully obtained for 448 individuals from 14 populations (Table 1). Sequence comparison revealed 139 distinct haplotypes, defined by 95 polymorphic sites (including 90 transitions, 15 transversions and no indels). Most of the haplotypes (90 out of 139, or 65%) were singletons. Of the remaining 49 haplotypes, 41 were shared among populations and eight were found in a single population (S3 Table). Only one haplotype (BiH001) was shared among all populations except for the Chesapeake Bay (CB). The number of haplotypes per population varied between nine (Black Sea) and 27 (Saltö), not considering North Carolina (NC) and Chesapeake Bay (CB), which had very small sample sizes (Table 1). The NC and CB populations also showed markedly lower haplotype diversity (0.29–0.52) and nucleotide diversity (0.0009–0.0012) compared to the other populations, which may be a result of small sample sizes (Table 1). Therefore, NC and CB populations were only included in sequence-based comparisons (e.g. Figs 1 and 2; where they were grouped as “Atlantic US”) but excluded from frequency-based analyses (pairwise Φ_ST_). Haplotype diversity for the 12 remaining populations ranged between 0.75–0.96. The lowest haplotype diversity out of these 12 populations was found in the northern Baltic populations—Umeå (0.85) and Öregrund (0.75)—as well as in the Caspian Sea (0.83) and Black Sea (BL: 0.82), and the highest was observed in the Estonian (0.96) and Japanese (0.95) populations (Table 1). Nucleotide diversity in the same 12 populations was relatively low and ranged from 0.0025 to 0.0069, with the lowest diversity found in the Black Sea (0.0025) and the highest diversity in Japan (0.0069) and Argentina (0.0063) (Table 1).

The geographic distribution of the 12 most frequent haplotypes (across the samples) revealed high haplotype diversity in most populations (Fig 1). The most common haplotypes (BiH001 and BiH002) were found in almost all sampled populations (S3 Table). The haplotype BiH001 was found in particularly high proportion within the Caspian Sea, Black Sea and in the northern Baltic Sea (Fig 1). The most common haplotype in the “Atlantic US” (BiH008) was only found in the Caspian Sea and Black Sea, suggesting a possible introduction to these areas from the Atlantic US. However, haplotype diversity was considerably higher within the Caspian Sea and Black Sea, indicating the possibility of multiple sources contributing to the populations found in these regions, although small samples sizes from the Atlantic US populations limits the possibility to draw conclusions on this issue.

**Fig 3:**
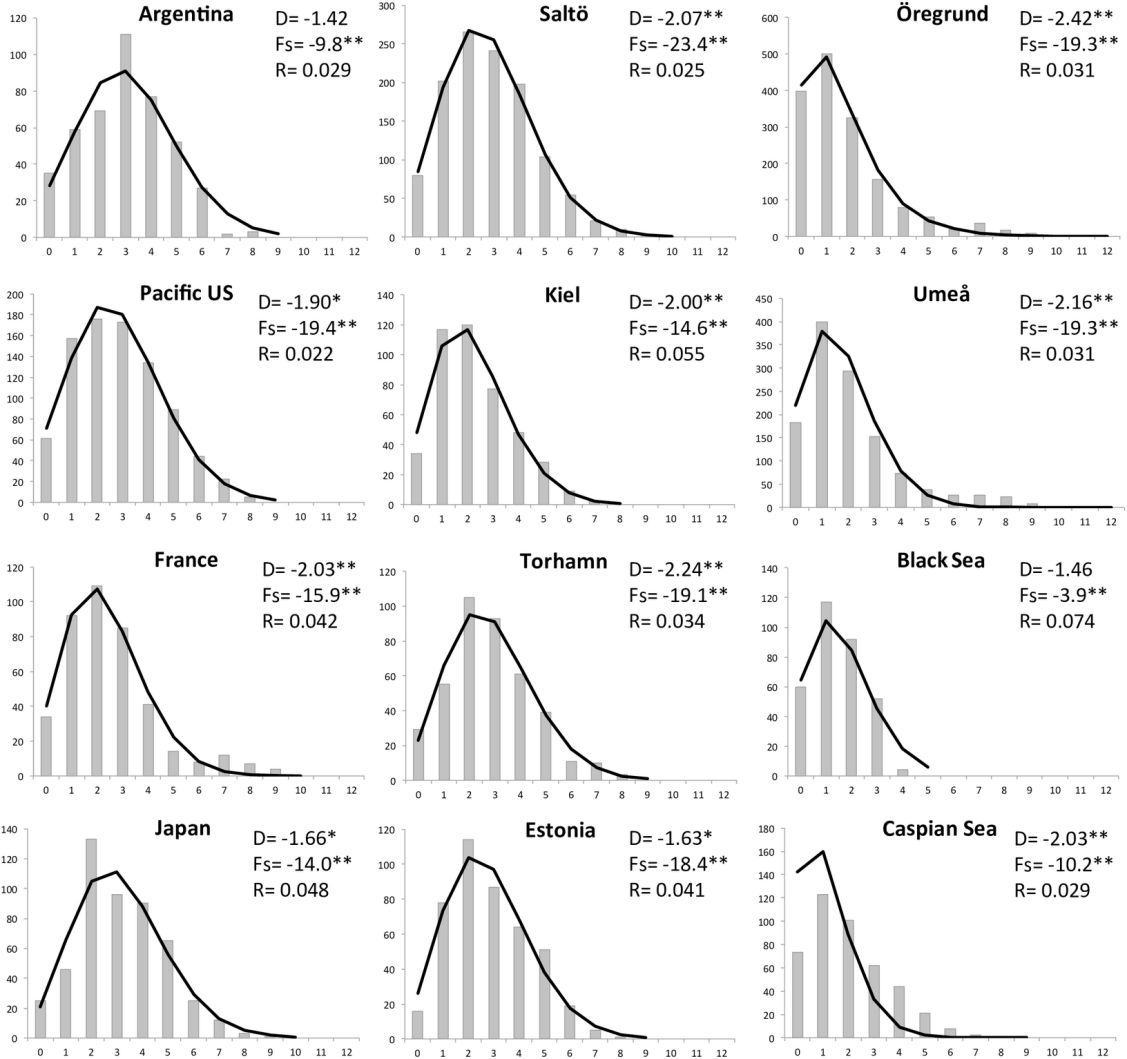
Mismatch distributions based on COI sequences from *B*. *improvisus* populations. Observed mismatch distribution (bars) and expected mismatch distributions under the sudden expansion model (solid line) of mtDNA COI sequences for each population. *D* = Tajima’s D, *F*_S_ = Fu’s *F*_S_, R = raggedness value. Significance is presented as *: P = 0.01–0.05, **: P < 0.01. The populations are sorted according to the pattern of mismatch distribution, with populations displaying more diverged haplotypes (2–4 mismatches) to the left, and populations with less differentiated haplotypes (1–2 mismatches) to the right.

The maximum likelihood phylogenetic analysis showed a shallow topology with no support for division of haplotypes into different clades (most bootstrap values were zero; see S3 Fig). The minimum spanning tree revealed a star-like shape with four main haplotypes surrounded by many unique haplotypes, mostly differing by no more than one or two mutation(s) (Fig 2). The star-like shape of the minimum spanning tree was congruent with the high haplotype diversity and low nucleotide diversity described above for most populations, and this result suggests a recent demographic expansion of populations. The hypothesis of a recent demographic expansion in most populations is also supported by the tests of neutral evolution. For example, Tajima’s *D* showed significant negative values for all populations except the Black Sea population, and Fu’s *F*_S_ showed significant negative values for all analysed populations (Fig 3). Furthermore, the mismatch distribution analysis displayed unimodal distributions for all populations and the observed mismatch distributions were not significantly different from the expected distributions under a sudden expansion model (R: 0.02–0.07; p>0.05), indicating recent demographic expansions (Fig 3). The only indications of possible bottlenecks, based on haplotype and nucleotide diversity, were observed in the Black Sea, Caspian Sea and northern Baltic, where lower genetic diversity may reflect a founder effect. These areas are all relatively isolated, to which human-mediated dispersal on ships is the most likely route of colonisation. However, the BOTTLENECK tests based on microsatellite loci found no significant bottlenecks (see below).

### Microsatellite diversity within populations

A total of 443 individuals from 12 populations were successfully genotyped at four microsatellite loci. None of these loci were successfully amplified for the North Carolina (NC) and Chesapeake Bay (CB) populations despite multiple PCR trials. We identified 78 alleles in total (over all four loci), where the mean number of alleles per population ranged from 8 to 13 (S4 Table). No signs of large allele dropout or stutter were detected. There were no indications of linkage disequilibrium between the four loci (p > 0.6). Mean expected heterozygosity (*H*_E_) was similar in all populations, ranging from 0.76 in Kiel to 0.83 in Torhamn (Table 1). Allelic richness was highest in France (9.1) and Saltö (9.0) and lowest in the Black Sea (7.5) and Caspian Sea (7.7). All four loci deviated from Hardy Weinberg Equilibrium (HWE) and three out of these four loci (B4, E29 and E42) showed considerable heterozygote deficiency (S2 Table, S4 Table and S5 Table). Their mean null allele frequencies were estimated to 0.39, 0.23 and 0.48, respectively (S5 Table). However, we found similar patterns of genetic differentiation using the locus C103 (almost in HWE), as when using the three loci with high heterozygote deficiency (S6 Table, S4 Fig). Despite indications of bottlenecks based on low nucleotide diversity (COI) in some populations, the BOTTLENECK analyses for microsatellites revealed no significant demographic bottlenecks (identified as excess of heterozygotes) using the Wilcoxon tests (P>0.05) with a FDR correction.

### Genetic structure among populations

Population genetic differentiation (pairwise Φst) based on COI data varied from -0.009 (Torhamn-Kiel) to 0.167 (France-Black Sea), with an overall average of 0.039 ±0.005 (SE) (Table 2). Based on COI data, 32 out of 66 population pairs were significantly differentiated from each other (Table 2). France showed significant differentiation from most populations, except from Kiel (KL) and Pacific US (PU). Both Argentina (AR) and Pacific US (PU) were only differentiated from the semi-enclosed, low salinity seas: Caspian Sea (CS), Black Sea (BL) and northern Baltic (UM and OR). The Caspian Sea (CS) and Black Sea (BL) were not significantly differentiated from each other. The Black Sea was differentiated from all other populations (Φst = 0.043–0.192) whereas the Caspian Sea (CS) was differentiated from most other populations, except the southern Baltic (SA, KL and TO) and Japan (JP). Populations in the northern Baltic (UM and OR) were not differentiated from each other (Φst = -0.007, p>0.84). Similarly, the southern Baltic-Skagerrak populations (SA, KL, TO, ES) were not differentiated from each other (Φst <0.003, p>0.3). However, the northern Baltic was differentiated from some populations in the southern Baltic (ES and KL; Φst = 0.027–0.03, p<0.014), and was highly divergent from populations in the Atlantic and Skagerrak (AR, FR and SA, Φst = 0.044–0.127, p<0.0001).

**Table 2:** Pairwise population genetic differentiation between populations of the barnacle *B*. *improvisus*, based on mtDNA and microsatellites.

	Argentina	France	Salto	Kiel	Torhamn	Estonia	Oregrund	Umea	BlackSea	CaspianSea	Japan	Pacific US
Argentina	-	0.034	0.024	0.008	0.019	0.027	0.019	0.014	0.004	-0.002	0.001	0.008
France	0.029	-	0.006	0.016	0.013	0.007	0.008	0.011	0.022	0.019	0.025	0.009
Salto	-0.003	**0.028**	-	0.014	0.011	0.008	0.012	0.009	0.024	0.015	0.020	0.017
Kiel	0.013	0.028	-0.004	-	0.008	0.006	0.005	0.007	0.018	0.013	0.012	0.006
Torhamn	0.024	**0.040**	-0.001	-0.009	-	-0.004	0.002	0.000	0.025	0.021	0.024	0.017
Estonia	0.017	**0.037**	0.003	-0.005	0.004	-	-0.001	0.000	0.026	0.023	0.026	0.017
Oregrund	**0.106**	**0.127**	0.047	**0.027**	0.001	0.044	-	0.003	0.019	0.017	0.015	0.010
Umea	**0.097**	**0.124**	**0.044**	**0.027**	0.005	**0.030**	-0.007	-	0.025	0.014	0.014	0.015
BlackSea	**0.106**	**0.167**	**0.062**	**0.059**	**0.043**	**0.088**	**0.047**	**0.054**	-	-0.002	0.024	0.016
CaspianSea	**0.041**	**0.098**	0.016	0.010	-0.002	**0.035**	**0.020**	**0.027**	0.002	-	0.005	0.005
Japan	0.017	**0.085**	0.007	0.038	0.025	0.019	**0.079**	**0.059**	**0.076**	0.032	-	0.000
Pacific US	-0.006	0.018	0.000	0.011	0.018	0.009	**0.091**	**0.079**	**0.111**	**0.052**	0.018	-

Below diagonal: pairwise Φ_ST_ (COI) based on Tamura and Nei (1993) genetic distances. Above diagonal: Pairwise *F*_ST_ based on microsatellite data (null-allele corrected). Numbers in bold are significant according to FDR, P-value = 0.03). P-values for null allele corrected data could not be obtained due to low number of loci (see methods).

Estimates of microsatellite pairwise *F*_ST_, after null allele corrections (mean *F*_ST_ = 0.013±0.001 SE), were similar to uncorrected estimates (*F*_ST_ = 0.015±0.001) (S6 Table). Hence, the null allele corrected data were used in the population structure analyses. The microsatellite data showed patterns of pairwise differentiation similar to the mtDNA COI results, albeit with slightly lower differentiation–pairwise *F*_ST_ varied from -0.002 (Black Sea-Caspian Sea) to 0.034 (France-Argentina) with an average of 0.013 ±0.001 (Table 2).

**Fig 4:**
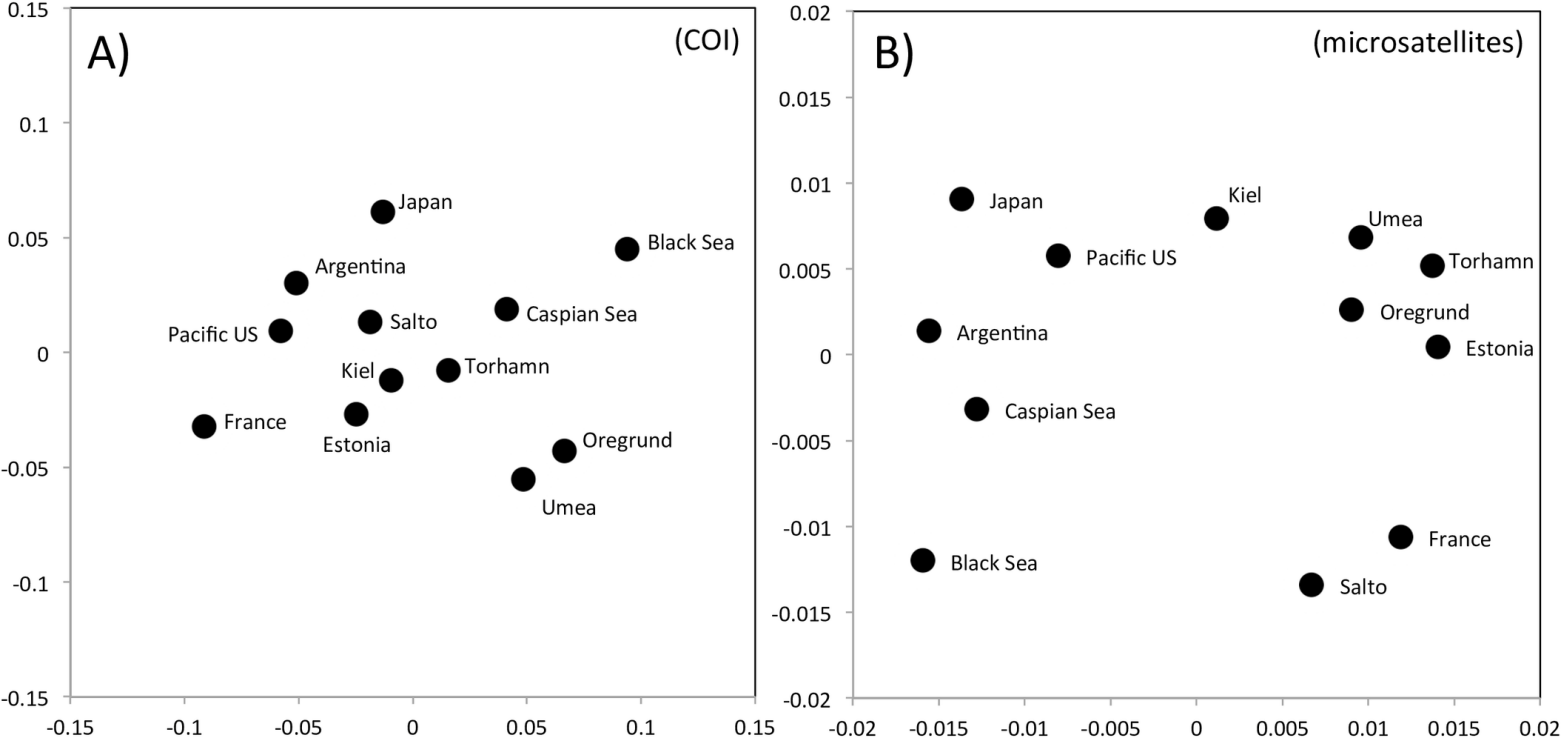
Non-metric multidimensional scaling plot (MDS) based on Slatkin’s linearized distances among populations of *B*. *improvisus*. A) MDS based on mtDNA (Φ_ST_): stress: 0.064; B) MDS based on microsatellites (F_ST_): data including all four loci with null allele correction, stress: 0.11 (stress values over 0.2 suggest that the precision in representing the relationships among populations is limited).

Both the MDS plots, visualising similarity between populations in 2-dimensions based on microsatellites and mtDNA, grouped the Baltic populations together, although the northern Baltic (UM and OR) and southern Baltic (TO, ES, KL) were separated into two clusters only in the mtDNA data (Fig 4). Saltö (SA) grouped together with France based on microsatellites, whereas the COI data placed Saltö (SA) with the other southern Baltic populations (KL, TO, ES), as well as partly with Argentina (AR) and the Pacific populations (PU, JP) (Fig 4). France was clearly different from most populations, especially from the northern Baltic (UM, OR) and Asian populations (CS, BL and JP), probably due to the high frequency of unique COI haplotypes present in this population (Table 1). Both microsatellites and mtDNA COI grouped the Black Sea and Caspian Sea together, where the Black Sea was more differentiated from the rest of the populations.

The AMOVA based on COI sequences from the Baltic-Skagerrak populations, corroborated the Φst pattern of differentiation within the Baltic, and showed that 3.7% of the variation was partitioned between the northern Baltic and southern Baltic-Skagerrak (*F*_CT_ = 0.037; p<0.0001), whereas slightly less variation (2.1%) was explained when separating Saltö from the Baltic populations (*F*_CT_ = 0.021; P<0.0001) (S7 Table). No variation between populations within these groupings was detected in either analysis (*F*_SC_ < 0.009, p>0.07) (S7 Table). In contrast, the Fst values based on microsatellite data (Table 2), showed that Saltö (SA) was more differentiated from the other Baltic populations (Fst: 0.008–0.014) than the other Baltic populations were from each other (Fst: -0.004–0.008), indicating a potential dispersal barrier at the entrance to the Baltic Sea. This pattern has been observed for many other Baltic species [61,62].

### Inference of invasive history based on modelling

The results from the DIYABC modeling of the potential invasive history of *B*. *improvisus*, showed that scenarios 3 and 4 received very weak support based on the posterior probabilities using logistic regression of COI summary statistics (S5A Fig). This may suggest that Europe is an unlikely native area (as in scenario 4), and that the invasion into the Black Sea and the Caspian Sea occurred from the Pacific region rather than from the eastern Atlantic (as in scenarios 1 and 2). The picture was slightly different when scenario probabilities were based on the observed microsatellite loci. Here, scenario 2 received very weak support where the US Pacific was the native area (S5B Fig), while scenario 3 with Argentina as the native area received the strongest support. A possible consensus based on both COI and microsatellite markers, is that scenarios with Argentina (scenarios 1 and 3) representing the native area received the strongest support. However, the rather different results for the COI and microsatellite markers indicate that the invasion structure is weak, again pointing to that mixing through shipping has been important. For the more detailed scenarios of the invasion into the Baltic Sea both markers gave weak support for the hypothesis of a Ponto-Caspian introduction (S6 Fig).

Posterior estimates of the time for introductions generally point to a recent expansion in the range of 100 to 1000 generations. Surprisingly, the COI data produced slightly more recent split times than the microsatellite data. These recent split times are also consistent with a global mixing of genotypes through the increase of shipping activity the past 200 years.

### Comparison of gene flow to geographical distance and oceanographic connectivity

**Fig 5:**
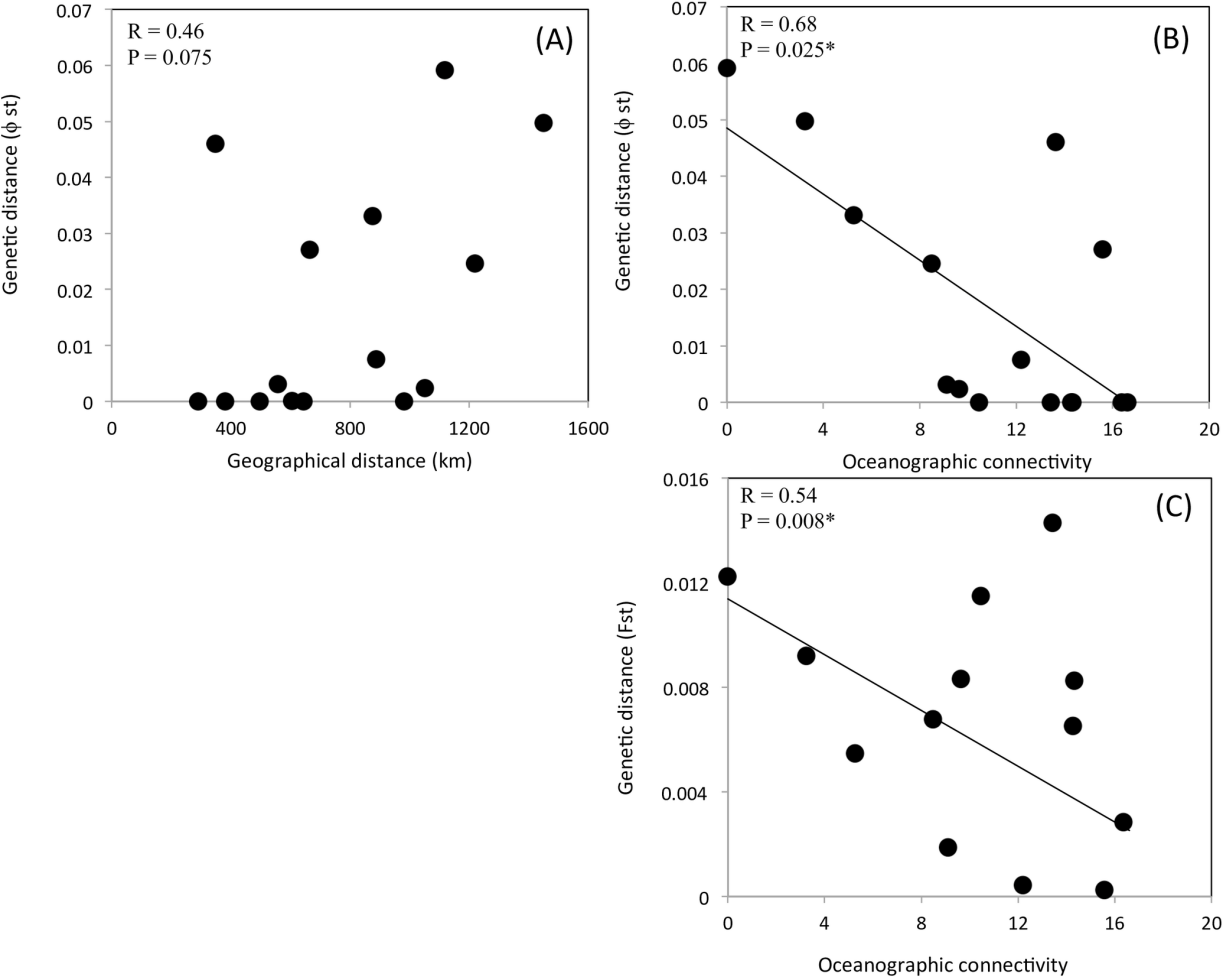
Correlating pairwise genetic distances between *B*. *improvisus* populations to geographical distance and oceanographic connectivity. Pairwise genetic distance (Φ_ST_, COI) for populations of *B*. *improvisus* in the Baltic Sea plotted as a function of (a) closest geographical shipping distance (km) (Mantel test: R = 0.46, p = 0.075); (b) minimum oceanographic connectivity between sampling sites; (c) standardised and null allele corrected pairwise genetic distance ((*F*_ST_/1-*F*_ST_) in microsatellites plotted as a function of minimum oceanographic connectivity between sampling sites.

On a global scale, there was a significant pattern of isolation by geographical distance (defined as “closest shipping distance”) for microsatellites (R = 0.37, P = 0.005), but not for mitochondrial DNA (R = 0.032, P = 0.49). In the Baltic-Skagerrak region, microsatellites showed no isolation by distance (IBD) (R = 0.30, P = 0.13), and for mitochondrial DNA there was a nearly significant IBD (R = 0.46, P = 0.075) (Fig 5A). Oceanographic connectivity, on the other hand, seemed to better explain patterns of genetic differentiation in the Baltic-Skagerrak region where both microsatellites (R = -0.54, P = 0.008) and mitochondrial DNA (R = -0.68, P = 0.025) revealed a significant correlation between genetic differentiation and (minimum) connectivity between sampling sites (Fig 5B and 5C).

## Discussion

### High levels of genetic diversity in *B*. *improvisus*

In this first study investigating global population genetic structure of the widely distributed barnacle *B*. *improvisus*, we found high levels of genetic diversity in both mitochondrial and nuclear markers in most populations (h = 0.75–0.96, Ar = 7.5–9.1). These levels are also high when compared to several other invertebrate species with similar invasive backgrounds, such as the tunicate *Botrylloides violaceus* (h = 0.0–0.61, Ar = 3.0–4.4) [109], or the globally invasive crab *Carcinus maenas* (h = 0.3 vs. 0.8; Ar = 3.1 vs. 4.4, for introduced vs. native populations) [25]. However, a recent global phylogenetic study on a closely related barnacle *Amphibalanus amphitrite* [24] revealed similar levels of genetic diversity in COI (h = 0.83–0.97).

Previous phylogeographic studies on barnacles have typically investigated regional genetic structure connected to present dispersal patterns [110,111] or questions related to historical post-glacial colonisation events [30,112]. Few genetic studies have aimed to assess the source of origin and colonization history of invasive barnacles (but see [24,113,114]). In the study by Geller et al. [113], they used mitochondrial and nuclear genes to successfully identify the source of introduction of several *Balanus glandula* populations along the Pacific US coast. However, the genetic diversity within populations was very low due to the recent invasion (1970’s), and no evidence of multiple sources was detected. In contrast, Chen et al. [24] found high genetic diversity in COI in the barnacle *A*. *amphitrite*, where sequences were divided into three distinct clades (with a 4% divergence between clades) indicating historical isolation between populations or possibly a cryptic species complex. In comparison, we found high genetic diversity within all *B*. *improvisus* populations, but could not identify any phylogenetic clades. Furthermore, no clear colonisation routes were identified, probably because high global shipping intensity has resulted in high admixture between populations.

Microsatellite results revealed very high heterozygote deficiencies relative to Hardy-Weinberg expectations (HWE) in three out of four loci (S4 Table, S5 Table), which may be due to several different reasons including technical artefacts such as null alleles (alleles that are not amplified due to e.g. high sequence variability at primer sites) and/or biological reasons such as Wahlund effects, inbreeding and self-fertilization. *B*. *improvisus* is a simultaneous hermaphrodite, and self-fertilization has been suggested to occur [115,116], although there are no robust experimental evidence of selfing (only anecdotal observations from field collected samples) and this possibility still requires testing. Furthermore, locus C103 showed no signs of null alleles, suggesting locus-specific issues rather than inbreeding. The heterozygote deficiencies, along with the overall low success in developing polymorphic microsatellites (4 out of 108 tested loci), are possibly due to primer mismatch (resulting in null alleles) associated with the high nucleotide diversity that we observe in the on-going genome sequencing project for *B*. *improvisus* (www.cemeb.science.gu.se/research/imago-marine-genome-projects). Heterozygote deficiency is commonly observed in some invertebrate groups [26,27] and the causes and consequences for interpreting genetic structure has been widely discussed [90,117,118]. Although caution should be taken for over-interpreting our results based on microsatellites, especially since only four loci were used, several findings corroborate our results: First, we found similar patterns of genetic differentiation using the locus C103 almost in HWE, as when using the three loci with high heterozygote deficiency (S4 Fig); Second, there were only slight differences in the pattern of genetic differentiation between the null allele corrected data and the non-corrected data, based on all four loci (S6 Table); Finally, the microsatellites and the mtDNA gave overall congruent results, that also correlated with seascape connectivity (Table 2).

### Origin and invasion history of *B*. *improvisus*

It is commonly stated in the literature that *B*. *improvisus* originates from the Atlantic coast of North and/or South America (e.g. [41,43,56]), although there is no clear evidence to support this. We were not able to confirm this hypothesis based on our present data; the Atlantic US populations (CB and NC) had low genetic diversity and were highly differentiated from all other populations (see Fig 1), two characteristics that are not expected of an original "source" population [119]. Furthermore, the complete amplification failure of the microsatellite loci for the Atlantic US populations indicates high divergence from the other populations. However, it is likely that the small sample size (N = 14) from the Atlantic US population has biased our data in this region, and it is thus impossible to draw conclusions about the hypothesis of an Atlantic North-American origin of *B*. *improvisus*. However, we should note that we sampled and sequenced 128 putative "*B*. *improvisus*" individuals from the Chesapeake Bay (CB) and North Carolina (NC), but that most of these were later most likely identified as *Balanus subalbidus* based on their highly divergent COI sequences and additional sequencing of other individuals that were identified as *B*. *subalbidus* based on morphology. *B*. *subalbidus* is an estuarine barnacle species with similar morphology to *B*. *improvisus*, especially as juveniles [42]. The high frequency of *B*. *subalbidus* in our samples indicates that *B*. *subalbidus* may be more common in the Chesapeake Bay and Neuse River area (North Carolina), than previously acknowledged [42]. Barnacles are known to cause competitive exclusion [120,121], however, nothing is known about the competitive interactions between *B*. *subalbidus* and *B*. *improvisus*. If *B*. *improvisus* has indeed been partly out-competed by *B*. *subalbidus* in low salinities, then the remaining small population sizes of *B*. *improvisus* could result in partial isolation and reduced genetic diversity. Furthermore, nothing is known about possible hybridization between *B*. *improvisus* and *B*. *subalbidus*, where e.g. introgression also could explain the mismatch between the amplification of mtDNA and microsatellites for these samples. Clearly, more studies are required to evaluate this possibility.

In contrast to the north Atlantic US population, there is more support for the Argentinian (southwest Atlantic) population being part of the native region for *B*. *improvisus*, based on relatively high haplotype and nucleotide diversity (Table 1), as well as showing no signs of recent expansions (Fig 3). Furthermore, most haplotypes that were found in the Argentinian population were also found in other populations globally (Fig 1). The ABC modelling also suggested that *B*.*improvisus* originates from America (S1 Fig, S5 Fig). To confirm this hypothesis, however, additional populations from the area (including both north and south America) must be sampled and compared.

Being a common fouling species on ship hulls, *B*. *improvisus* has probably been transported between coasts for many centuries, even before the species first was described by Darwin in 1854. Therefore, the early descriptions of *B*. *improvisus* from the Atlantic American coast may not provide evidence of the true origin of *B*. *improvisus*. The fact that many introduced populations had a high proportion of private haplotypes (i.e. haplotypes not shared with other populations, including potential native regions), suggests that we may have missed several native populations in our sampling for this study, and thereby the extent of native genetic diversity could be greatly underestimated. For example, the French population displayed a remarkably high proportion (34%) of unique haplotypes (Table 1). Alternatively, the notionally native North-American population(s) may have experienced a recent bottleneck, possibly due to competition with other barnacle species such as *B*. *subalbidus*; such a bottleneck may have drastically reduced the native genetic diversity to levels below those of the invasive populations. The spatial and temporal resolution of the genetic and historical data is as yet inadequate to permit a coherent conclusion about the native region.

### Evidence of bottlenecks and recent demographic expansions

A common assumption regarding introduced populations has been that they should experience loss of diversity relative to native sources because of founder effects and post-introduction demographic bottlenecks [61,119]. For example, Flight et al. [112] found higher haplotype diversity and allelic richness in European native populations of the barnacle *S*. *balanoides* (*h* = 0.99, Ar = 15.7) compared to North American (introduced) populations (h = 0.86, Ar = 12.3). However, many recent studies have shown that introduced populations show little reduction in genetic diversity, and that some may even display higher genetic diversity than in the native range, caused by multiple introductions from different source populations [113,122].

In our study, we found high genetic diversity in almost all populations (except “Atlantic US”; *h* = 0.75–0.96, Ar = 7.5–9.1). This is likely the result of multiple introductions from different sources, with large founding populations and/or potential admixture, e.g. [122,123], exemplified here by fouling organisms such as barnacles, that often form dense populations on ship hulls and are transported between harbours over large distances.

The mismatch distribution analysis as well as the neutrality tests, confirm expectations of a model of recent and sudden expansion of most populations (Fig 3). High haplotype diversity and low nucleotide diversity among haplotypes is another residual effect of recent evolutionary history. The only indications of possible bottlenecks, based on haplotype and nucleotide diversity, were observed in the Black Sea, Caspian Sea and northern Baltic, where lower genetic diversity may reflect a founder effect. These areas are all relatively isolated, to which human-mediated dispersal on ships is the most likely route of colonisation. However, based on the BOTTLENECK analyses for microsatellites, no significant demographic bottlenecks (identified as excess of heterozygotes) were detected using the Wilcoxon tests (P>0.05) with a FDR correction.

The main haplotype in the Atlantic US samples (BiH008) was also found in the Caspian Sea and Black Sea populations, supporting the idea that at least one introduction from Atlantic US to these regions has occurred (Fig 2). This introduction pathway has also been suggested for other species, e.g. *Mnemiopsis leyidi* [28]. However, the Caspian Sea and Black Sea also contained many other haplotypes not represented in the Atlantic US suggesting multiple introductions from different areas (notwithstanding the potential sampling error in the Atlantic US populations mentioned previously).

### The role of natural larval dispersal versus human-mediated dispersal

Colonisation history of fouling organisms is often complex, since human-mediated dispersal via maritime activities has occurred for several centuries. Especially during the past century, shipping intensity has increased dramatically, facilitating new invasions of non-indigenous species as well as potentially modifying population genetic structure in already established species [1]. Previous studies on other invasive invertebrate species have shown that patterns of long distance dispersal are correlated with human-mediated vectors [109,124].

The high genetic diversity that we observed in most populations including many shared haplotypes on a global scale, suggests that shipping and other anthropogenic activities have played an important role in shaping the current population genetic structure of *B*. *improvisus*. An interesting pattern, related to historical shipping, is the unexpectedly high similarity between Argentina (AR) and Saltö (SA) (Φst = -0.003). From the late 19th century to early 20th century there was intensive shipping activity between these regions: the small town Krokstrand (close to Saltö) in Sweden was a key port from which granite was shipped to several parts of the world during many decades, including Buenos Aires in Argentina where it was used to pave the streets of the city [125].

Another common source of introductions of non-native species is indirectly through aquaculture activities [126]. The high diversity and large proportion of private haplotypes observed in the French population in our study could partly be a result of multiple introductions from different source populations due to aquaculture. Although *B*. *improvisus* was observed in French waters already in 1872 [127], they were most likely also introduced along with the import of oysters (*Crassostrea gigas*) to French oyster farms in the 1960’s [128]. Other examples of species that were introduced to France through aquaculture are the widespread brown alga *Sargassum muticum* and the mollusc *Crepidula fornicata* [26,129]. Similar to our study, Dupont et al. [26] found high genetic diversity in introduced *C*. *fornicata* populations along the French coast, and suggested that these populations derived from several genetically diverse, but poorly differentiated source populations.

On a global scale, there was a pattern of isolation by geographical distance (defined as closest shipping distance) for microsatellites, but not for mitochondrial DNA. One possible explanation for this pattern is that most CO1 haplotypes were already present when global shipping began to translocate populations between places, thus resulting in a re-shuffling of “old” haplotypes that may poorly reflect geographic distance. Microsatellites on the other hand may reflect contemporary processes of gene flow and random drift; while it is also possible that new alleles can arise through mutations to produce an “isolation by distance” signal, it is unlikely that new mutations have caused this pattern (cf. [130]), given the short time for human-induced introduction (<200 years in most cases). In this context, it is interesting that Flight et al. [112] found similar differences between marker types for the barnacle *S*. *balanoides*.

Oceanographic connectivity seemed to better explain patterns of genetic differentiation at a regional scale (in the Baltic-Skagerrak region) where both microsatellites and mitochondrial DNA revealed a significant correlation with (minimum) connectivity between sampling sites (Fig 5B and 5C). The lack of genetic differentiation between two northern Baltic populations (UM and OR) as well as the low differentiation between the southern Baltic populations supports the idea that larval dispersal also plays an important role for further spreading of the species within the Baltic Sea (Table 2). Several studies on invertebrates with pelagic larval stages (including barnacles) have shown the importance of oceanographic connectivity for determining population structure [111,131,132]. However, oceanographic connectivity probably acts in synergy with human-mediated dispersal. As an example, the differentiation of the northern Baltic populations (OR, UM) from the southern Baltic populations (KL, TO, ES) may be a combination of restricted larval dispersal as well as independent colonisation events via shipping from different source populations.

Notably, the AMOVA comparing groups of populations within the Baltic Sea (S7 Table) indicated that there is a stronger differentiation between the northern and southern Baltic-Skagerrak than between the Baltic populations and Saltö (Skagerrak). This is interesting, since the entrance to the Baltic Sea (Danish Belt/Öresund) is often considered a main breakpoint in genetic differentiation in many species, clearly separating Baltic and Atlantic populations [61]. However, selection for local adaptations to different environmental conditions (especially the salinity gradient in the Baltic), in addition to restricted gene flow due to oceanographic currents, may be an explanation for this pattern [61]. This could thus partly explain the different results observed in our study, since we used assumedly neutral markers (at least the microsatellites), reflecting mostly neutral processes including drift and gene flow, rather than selection. The role of natural selection caused by the strong environmental gradients in the Baltic Sea was recently investigated [48], however no populations from the northern Baltic Sea populations (latitude >60°N, salinity <5psu) were included, hence it remains to be studied in *B*. *improvisus*.

### Ecological and evolutionary implications for *B*. *improvisus*

That *B*. *improvisus* is a cosmopolitan species, successfully colonising many parts of the world, is not surprising. The species displays several life history traits that are associated with successful invaders including broad environmental tolerance (both to natural factors such as salinity, temperature and desiccation, but also to pollution; [9,48,133,134]. Broad tolerance may be a result of phenotypic plasticity, where an organism can maintain or adjust their phenotype when the environment changes, without genetic changes necessarily occurring [135]. An interesting comparison is the closely related barnacle *A*. *amphitrite* that has a narrower environmental tolerance [136] and displays a more structured population genetic pattern with several distinct clades [24], compared to *B*. *improvisus*.

Furthermore, *B*. *improvisus* displays multiple modes of dispersal (both through free-swimming larvae and fouling), has an extended spawning season and a low habitat selectivity at settlement [103], all of which facilitate colonisation of new areas at different scales. However, the species is often restricted to low salinity environments such as estuaries and coastal lagoons and harbours, suggesting that it may have a limited ability to successfully compete with other sessile invertebrates in high salinity environments. Estuaries are generally considered to be sources of diversification by adaptive divergence and spatial isolation [17]. Fluctuations in many abiotic factors, combined with isolation of habitat from other estuarine areas, often contributes to genetic structuring, enhancing effects of micro-evolutionary processes including drift, selection and gene flow [137]. However, in *B*. *improvisus*, intensive human-mediated gene flow probably counteracts these processes, resulting in the pattern of high diversity and little differentiation even on a global scale, as we found in this study. This high diversity and gene flow from multiple sources may have implications for the ability of *B*. *improvisus* to adapt to local conditions, compete with native species, spread to new areas as well as cope with future environmental change.

In conclusion, we found high genetic diversity in most populations, including both putatively native and introduced regions, indicating high gene flow between populations via shipping and other anthropogenic activities. Furthermore, mitochondrial and nuclear markers revealed similar patterns. Our results could not clearly identify the geographic origin of *B*. *improvisus*, but suggest that Argentina may at least be part of the native region. Further sampling of multiple populations in this area is required to clarify the pattern. Evidence of bottlenecks associated with founder events were only observed in the Caspian and Black Sea and in the northern Baltic Sea, although most populations showed genetic patterns consistent with recent expansion events. Oceanographic connectivity, rather than geographic distance, seems to explain regional genetic structure within the Baltic Sea, highlighting the importance of larval dispersal for further natural spreading of introduced species. Knowledge about different types of dispersal dynamics of non-native species is crucial to our understanding of both evolutionary aspects of colonisation as well as future management of biological invasions. The implications of the broad environmental tolerance and high dispersal capacity of *B*. *improvisus* for its ability to adapt to local conditions and tolerate future environmental changes remains to be elucidated.

## Supporting Information

S1 Fig Schematic figures of modeled scenarios of introductory history for *Balanus improvisus*. The first four scenarios (a, b) explore the most likely origin of *B*. *improvisus* on a global scale while the last two scenarios focus on two alternative colonization routes for the Baltic Sea. Abbreviations of population samples are: AR–Argentina, FR–France, SA–Saltö, BA–Baltic Sea, BL–Black Sea, CS–Caspian Sea, PU–Pacific US, JP–Japan, BA-S–southern Baltic Sea (KL, Torhamn, Estonia), BA-N–northern Baltic Sea (Öregrund, Umeå). See S1 Table for more geographic information.
(TIF)

S2 Fig PCA on summary statistics from the simulated datasets of the four global scenarios and the observed data (yellow) based on; (a) COI sequences and (b) microsatellite loci. (TIF)

S3 Fig Maximum likelihood phylogenetic tree based on all *B*. *improvisus* individuals in the study. The robustness of branches was tested with 100 bootstraps, and a COI sequence of *Balanus eburneus* was used as an out-group for this maximum likelihood analysis.
(PDF)

S4 Fig Multi-dimensional scaling plot (MDS) based on pairwise *F*_ST_ (not corrected for null alleles) for the different microsatellite loci. A) locus C103 separately; B) B4, E29 and E42 pooled. Although the loci B4, E29 and E42 showed high heterozygote deficiency, they display similar patterns to locus C103.
(TIF)

S5 Fig Posterior probability of the four ABC modeling scenarios for invasion of *B*. *improvisus* on a global scale. The figures are based on samples of (a) COI sequences, and (b) microsatellite loci.
(TIF)

S6 Fig Posterior probability of the two scenarios for introduction of *B*. *improvisus* into the Baltic Sea. The figures are based on samples of (a) COI sequences and (b) microsatellite loci.
(TIF)

S1 Table Information about the spatial and temporal sampling of DNA from *Balanus improvisus*. (TIF)

S2 Table Microsatellite primer information. The table contains locus name, repeat motif, primer sequence, universal tail name, MgCl_2_ (mM) = magnesium chloride concentration, Tm = melting temperature, Ta1 and Ta2 = annealing temperatures (see PCR protocols in methods); number of PCR cycles, Allele size range (bp); N = number of samples used to test microsatellites; Na = total number of alleles; Ho = observed heterozygosity; He = expected heterozygosity; F_IS_ = inbreeding coefficient; *F*_ST_ uncorr. = uncorrected average genetic distance between populations; *F*_ST_ corr. = null allele corrected average pairwise genetic distance between populations. Also included are the different tails and dyes added to the microsatellite primers (T1-D2, T2-D2, T2-D4).
(TIF)

S3 Table Haplotype list with haplotype frequencies (based on COI) for all *B*. *improvisus* populations. The populations are: Argentina (AR), France (FR), Saltö (SA), Kiel (KL), Torhamn (TO), Estonia (ES), Öregrund (OR), Umeå (UM), Black Sea (BL), Caspian Sea (CS), Japan (JP) and Pacific US (PU).
(PDF)

S4 Table Summary of microsatellite loci results for each population tested. Sample size (N), expected (He) and observed (Ho) heterozygosities, deviations from HWE (*F*_IS_) according to Weir & Cockerham (1984), number of alleles (A) and number of alleles per locus (Ar). Populations: Argentina (AR), France (FR), Saltö (SA), Kiel (KL), Torhamn (TO), Estonia (ES), Öregrund (OR), Umeå (UM), Black Sea (BL), Caspian Sea (CS), Japan (JP) and Pacific US (PU).
(TIF)

S5 Table Estimated null allele frequencies for microsatellites. The EM algorithm (Dempster et al. 1977) was used for each locus and *B*. *improvisus* population separately.
(TIF)

S6 Table Pairwise *F*_ST_ between *B*. *improvisus* populations based on four microsatellite loci. Below diagonal: Pairwise *F*_ST_ without correction for null alleles using ENA correction, FDR corrected significant differentiation in bold (FDR adjusted α_0.05_ = 0.0341); above diagonal: null-allele corrected pairwise *F*_ST_ between population. NB: p-values cannot be obtained in FreeNA when <5 loci.
(TIF)

S7 Table Analysis of molecular variance (AMOVA) results on *B*. *improvisus* Based on mtDNA (COI) data for populations in the Baltic Sea-Skagerrak region: UM = Umeå, OR = Öregrund, TO = Torhamn, KL = Kiel, ES = Estonia, SA = Saltö.
(TIF)
