# Supplementary figures and images for: The Story of a Hitchhiker: Population Genetic Patterns in the Invasive Barnacle Balanus(Amphibalanus) improvisus Darwin 1854
Source: PLoS One. 2016 Jan 28;11(1):e0147082. doi: 10.1371/journal.pone.0147082 (PMC4731558; doi:10.1371/journal.pone.0147082)

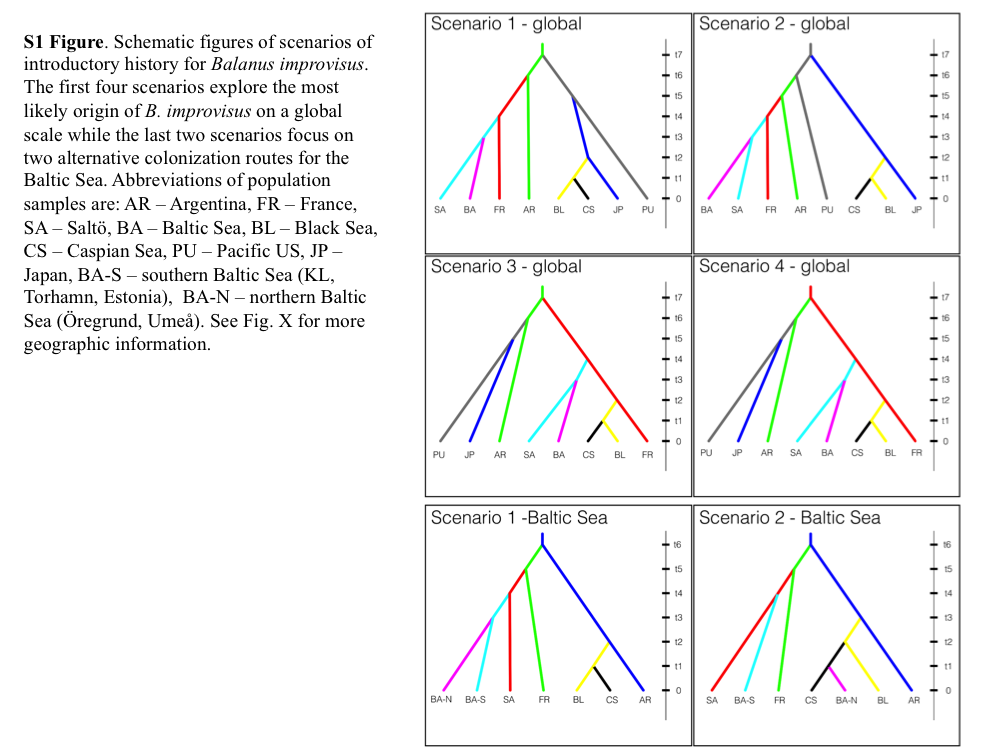

Supplement: S1 Fig — The first four scenarios (a, b) explore the most likely origin of B. improvisus on a global scale while the last two scenarios focus on two alternative colonization routes for the Baltic Sea. Abbreviations of population samples are: AR–Argentina, FR–France, SA–Saltö, BA–Baltic Sea, BL–Black Sea, CS–Caspian Sea, PU–Pacific US, JP–Japan, BA-S–southern Baltic Sea (KL, Torhamn, Estonia), BA-N–northern Baltic Sea (Öregrund, Umeå). See S1 Table for more geographic information. (TIF) [file pone.0147082.s001.tif]

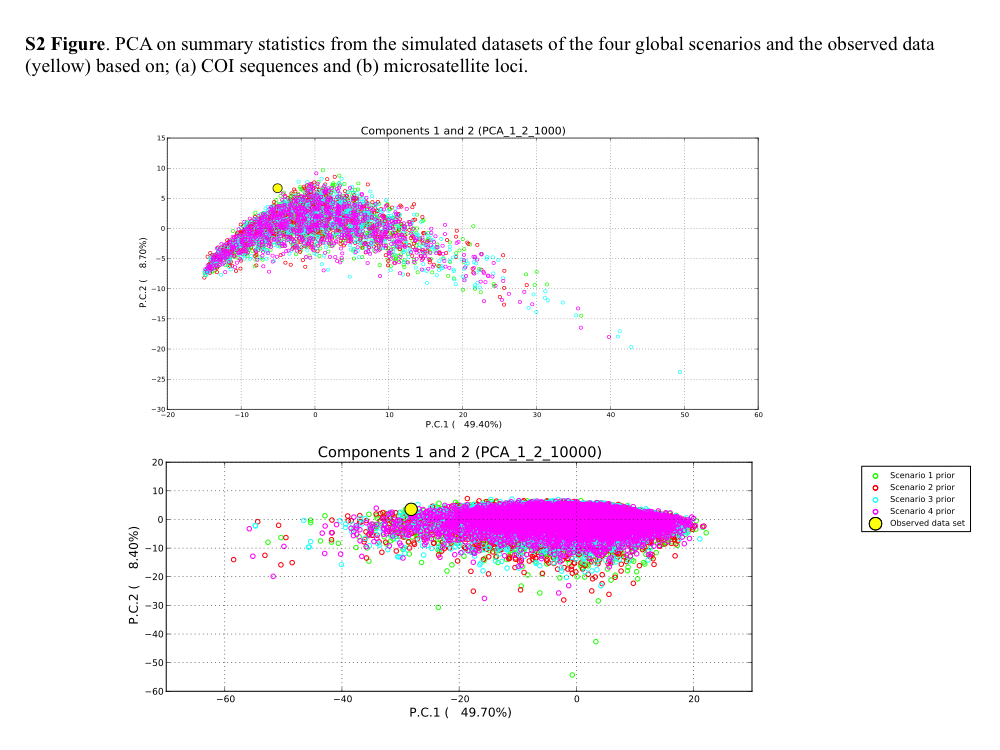

Supplement: S2 Fig — (TIF) [file pone.0147082.s002.tif]

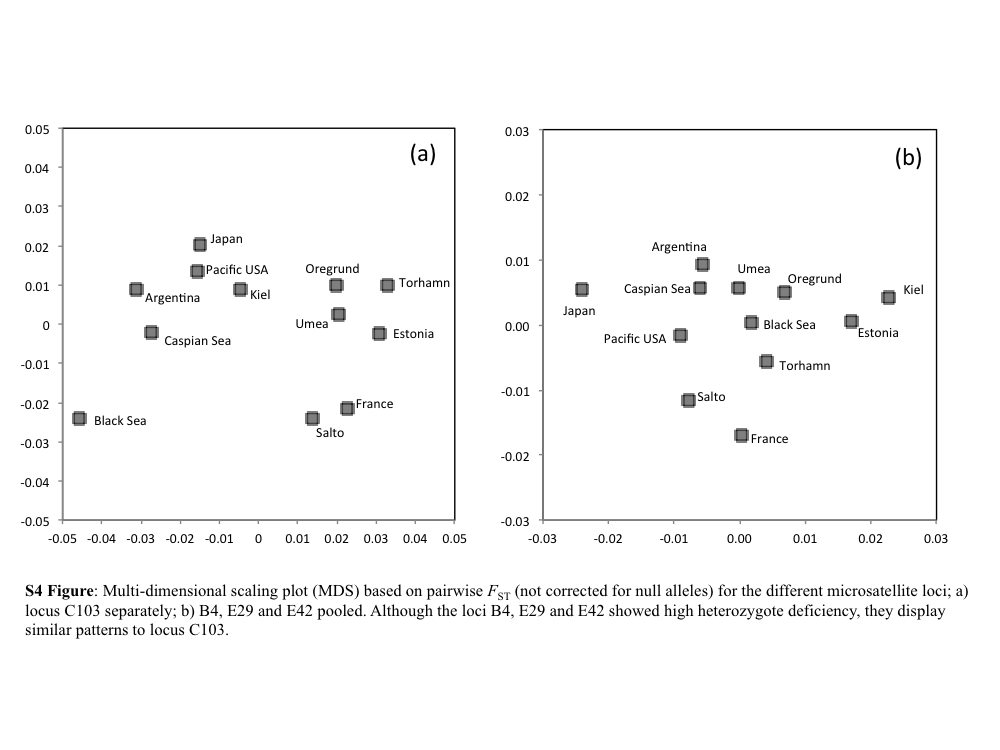

Supplement: S4 Fig — A) locus C103 separately; B) B4, E29 and E42 pooled. Although the loci B4, E29 and E42 showed high heterozygote deficiency, they display similar patterns to locus C103. (TIF) [file pone.0147082.s004.tif]

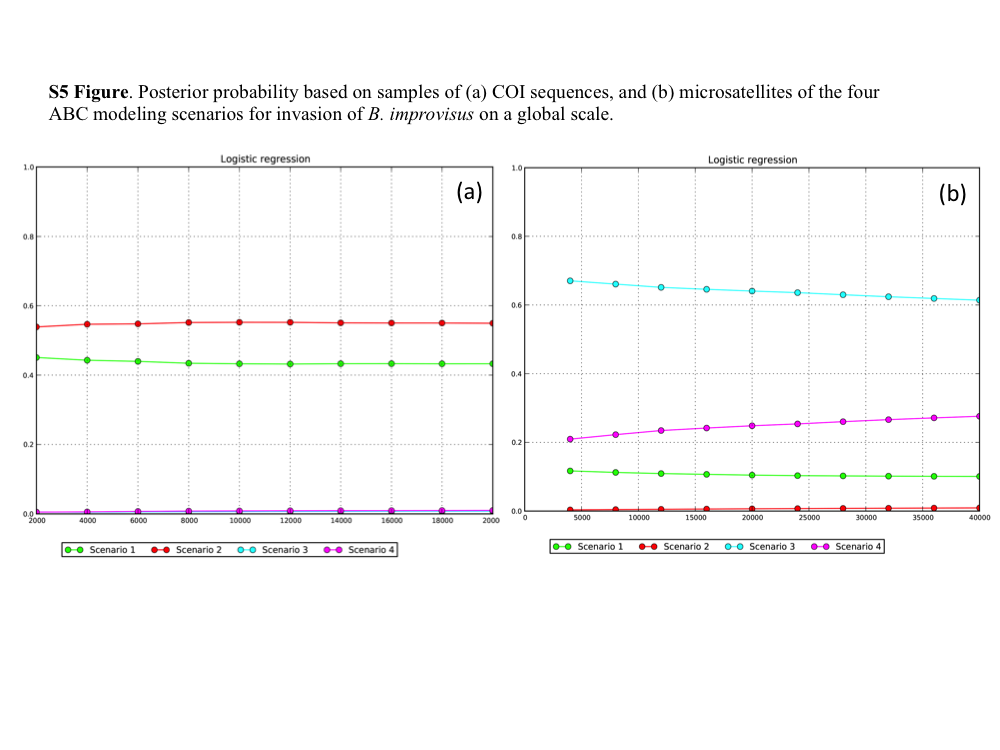

Supplement: S5 Fig — The figures are based on samples of (a) COI sequences, and (b) microsatellite loci. (TIF) [file pone.0147082.s005.tif]

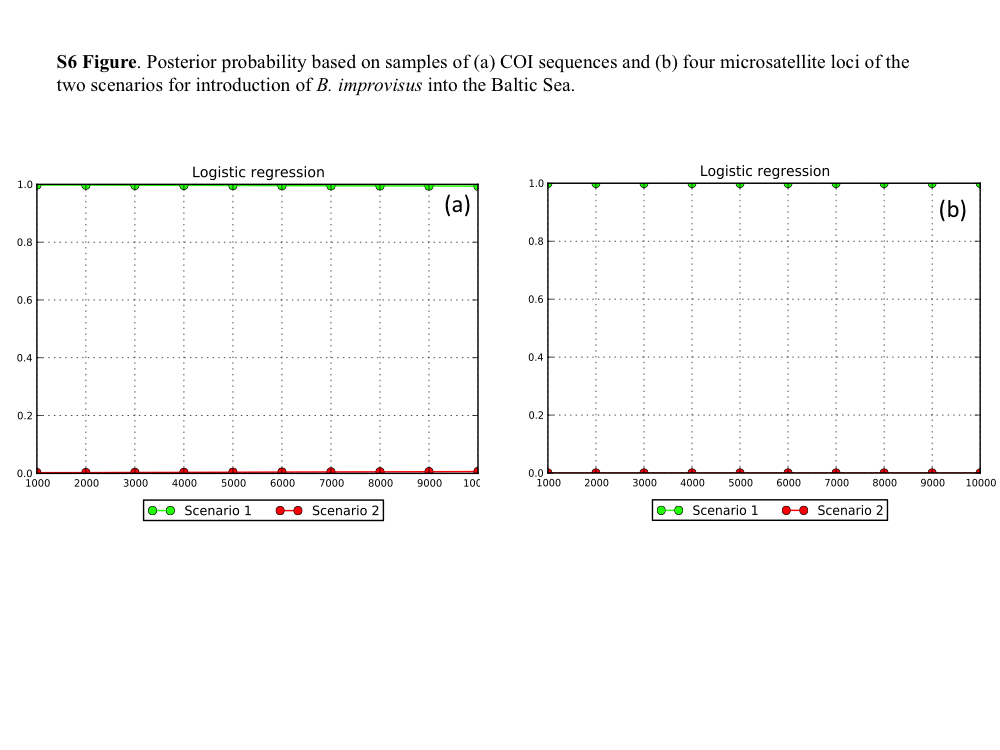

Supplement: S6 Fig — The figures are based on samples of (a) COI sequences and (b) microsatellite loci. (TIF) [file pone.0147082.s006.tif]

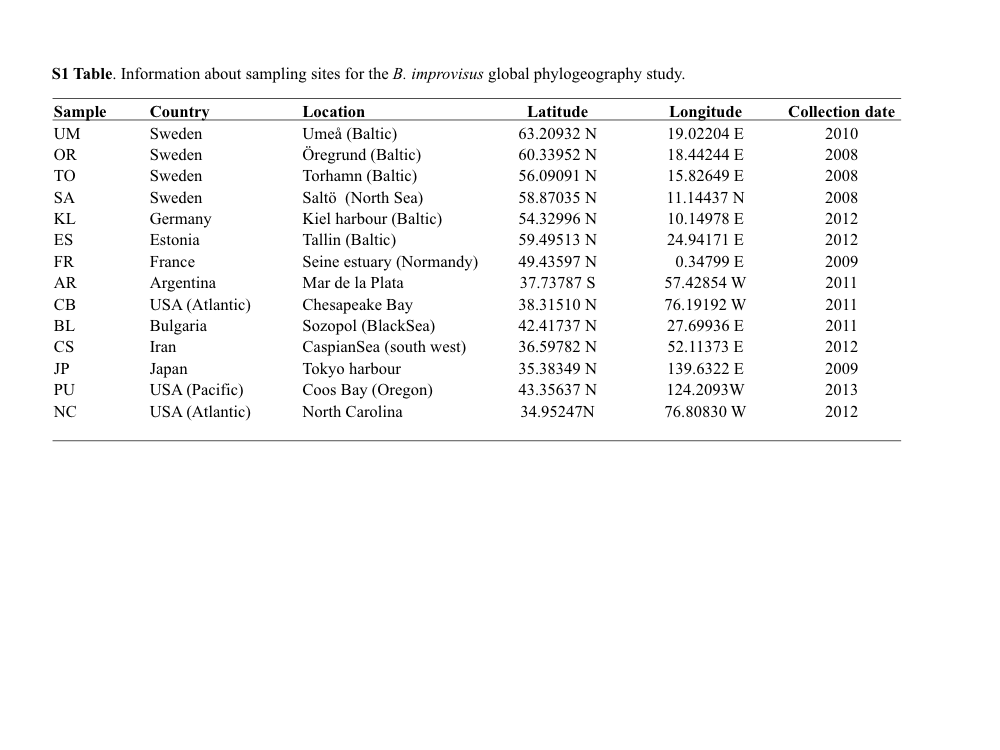

Supplement: S1 Table — (TIF) [file pone.0147082.s007.tif]

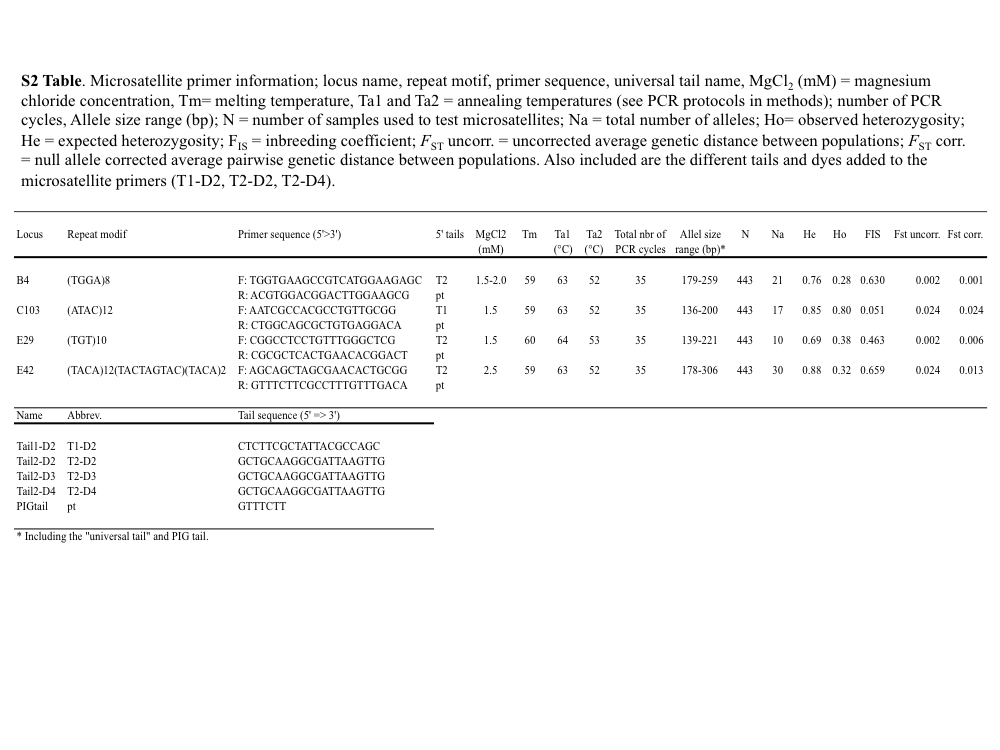

Supplement: S2 Table — The table contains locus name, repeat motif, primer sequence, universal tail name, MgCl2 (mM) = magnesium chloride concentration, Tm = melting temperature, Ta1 and Ta2 = annealing temperatures (see PCR protocols in methods); number of PCR cycles, Allele size range (bp); N = number of samples used to test microsatellites; Na = total number of alleles; Ho = observed heterozygosity; He = expected heterozygosity; FIS = inbreeding coefficient; FST uncorr. = uncorrected average genetic distance between populations; FST corr. = null allele corrected average pairwise genetic distance between populations. Also included are the different tails and dyes added to the microsatellite primers (T1-D2, T2-D2, T2-D4). (TIF) [file pone.0147082.s008.tif]

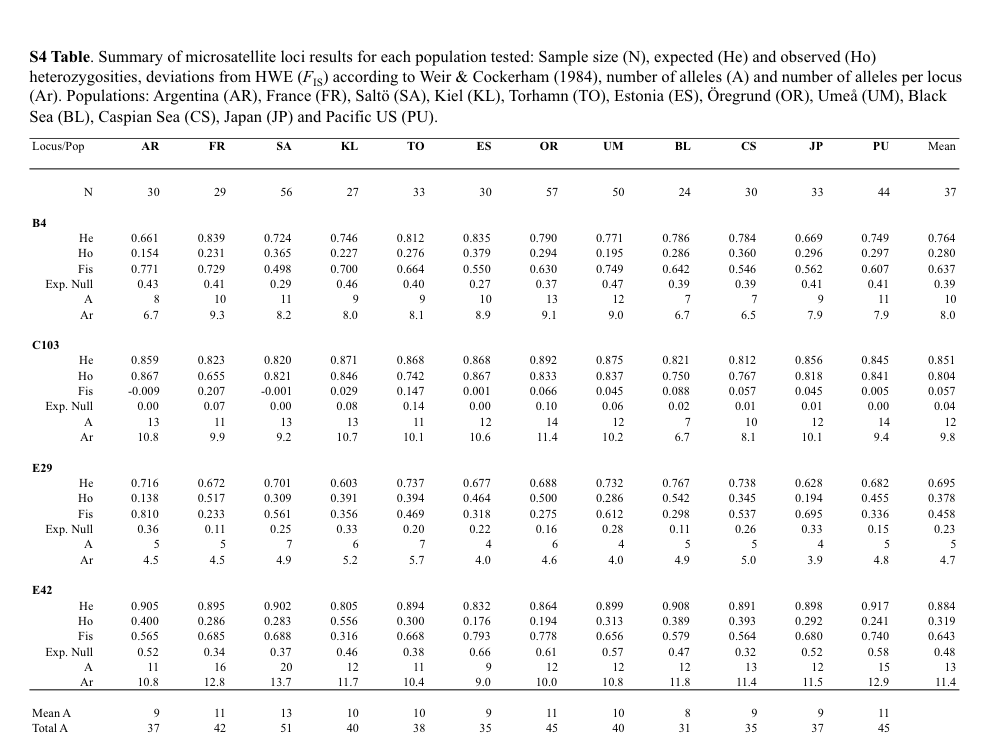

Supplement: S4 Table — Sample size (N), expected (He) and observed (Ho) heterozygosities, deviations from HWE (FIS) according to Weir & Cockerham (1984), number of alleles (A) and number of alleles per locus (Ar). Populations: Argentina (AR), France (FR), Saltö (SA), Kiel (KL), Torhamn (TO), Estonia (ES), Öregrund (OR), Umeå (UM), Black Sea (BL), Caspian Sea (CS), Japan (JP) and Pacific US (PU). (TIF) [file pone.0147082.s010.tif]

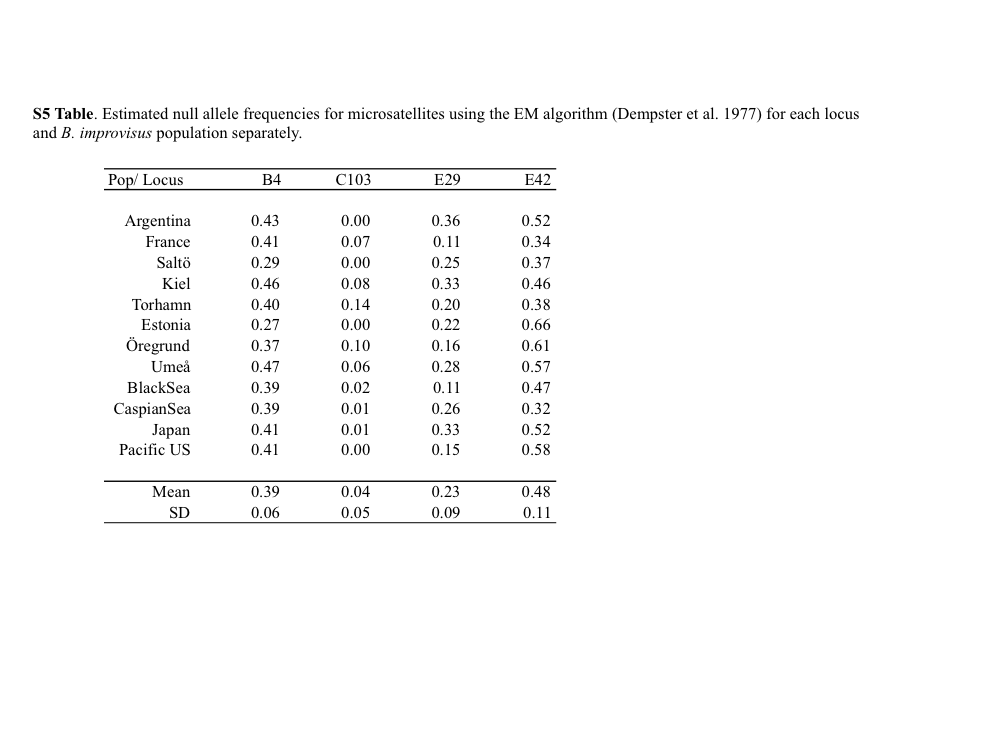

Supplement: S5 Table — The EM algorithm (Dempster et al. 1977) was used for each locus and B. improvisus population separately. (TIF) [file pone.0147082.s011.tif]

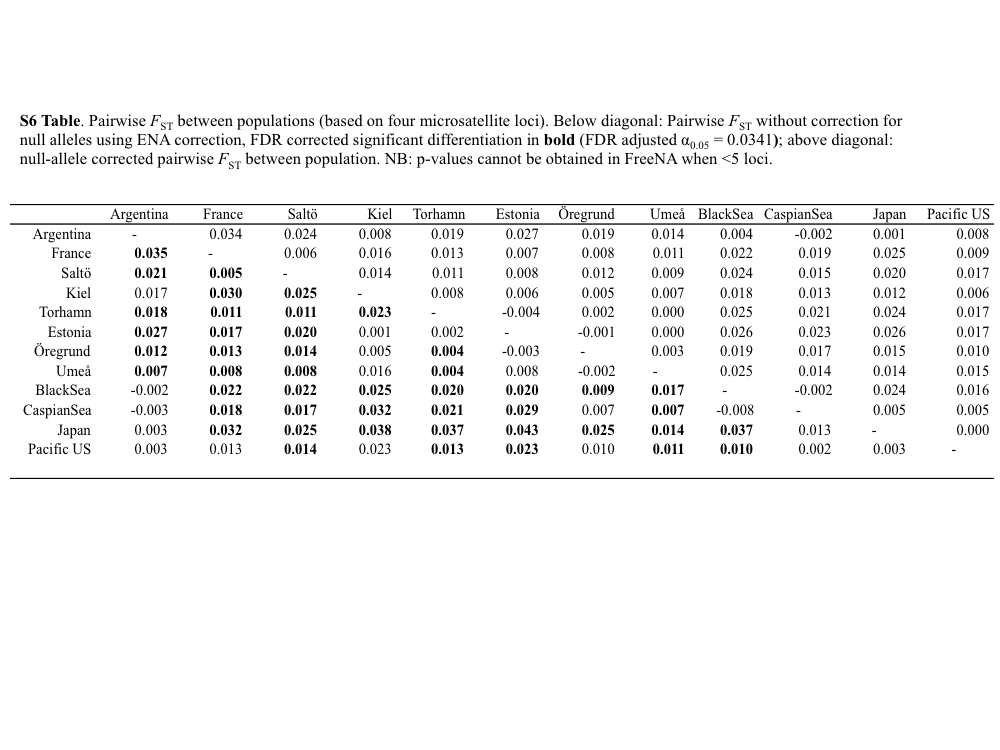

Supplement: S6 Table — Below diagonal: Pairwise FST without correction for null alleles using ENA correction, FDR corrected significant differentiation in bold (FDR adjusted α0.05 = 0.0341); above diagonal: null-allele corrected pairwise FST between population. NB: p-values cannot be obtained in FreeNA when <5 loci. (TIF) [file pone.0147082.s012.tif]

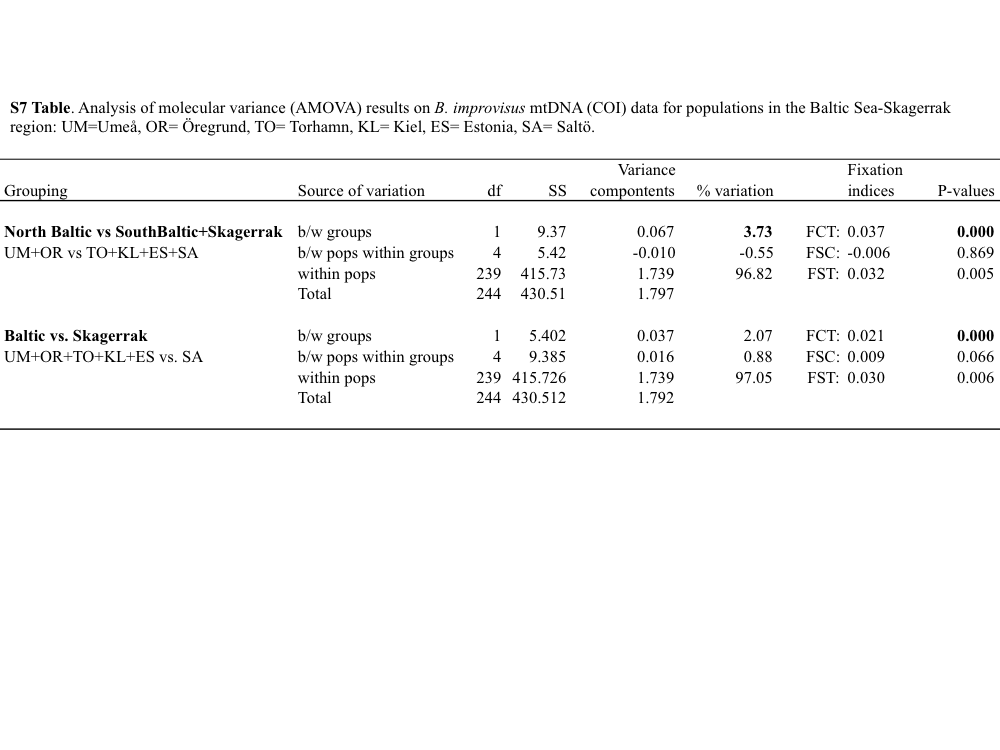

Supplement: S7 Table — Based on mtDNA (COI) data for populations in the Baltic Sea-Skagerrak region: UM = Umeå, OR = Öregrund, TO = Torhamn, KL = Kiel, ES = Estonia, SA = Saltö. (TIF) [file pone.0147082.s013.tif]
