# Supplementary material for: The Story of a Hitchhiker: Population Genetic Patterns in the Invasive Barnacle Balanus(Amphibalanus) improvisus Darwin 1854
Source: PLoS One. 2016 Jan 28;11(1):e0147082. doi: 10.1371/journal.pone.0147082 (PMC4731558; doi:10.1371/journal.pone.0147082)

S3 Figure. Maximum likelihood phylogenetic tree that was built with PHYML [76] implemented in GENEIOUS PRO. The HKY substitution model [80] with a gamma correction α = 1.015 and a proportion of invariant sites p-inv. = 0.691 was selected as the optimal model among those available in PHYML, according to JMODELTEST2. The robustness of branches was tested with 100 bootstraps, and a COI sequence of *Balanus eburneus* was used as an out-group for this maximum likelihood analysis.

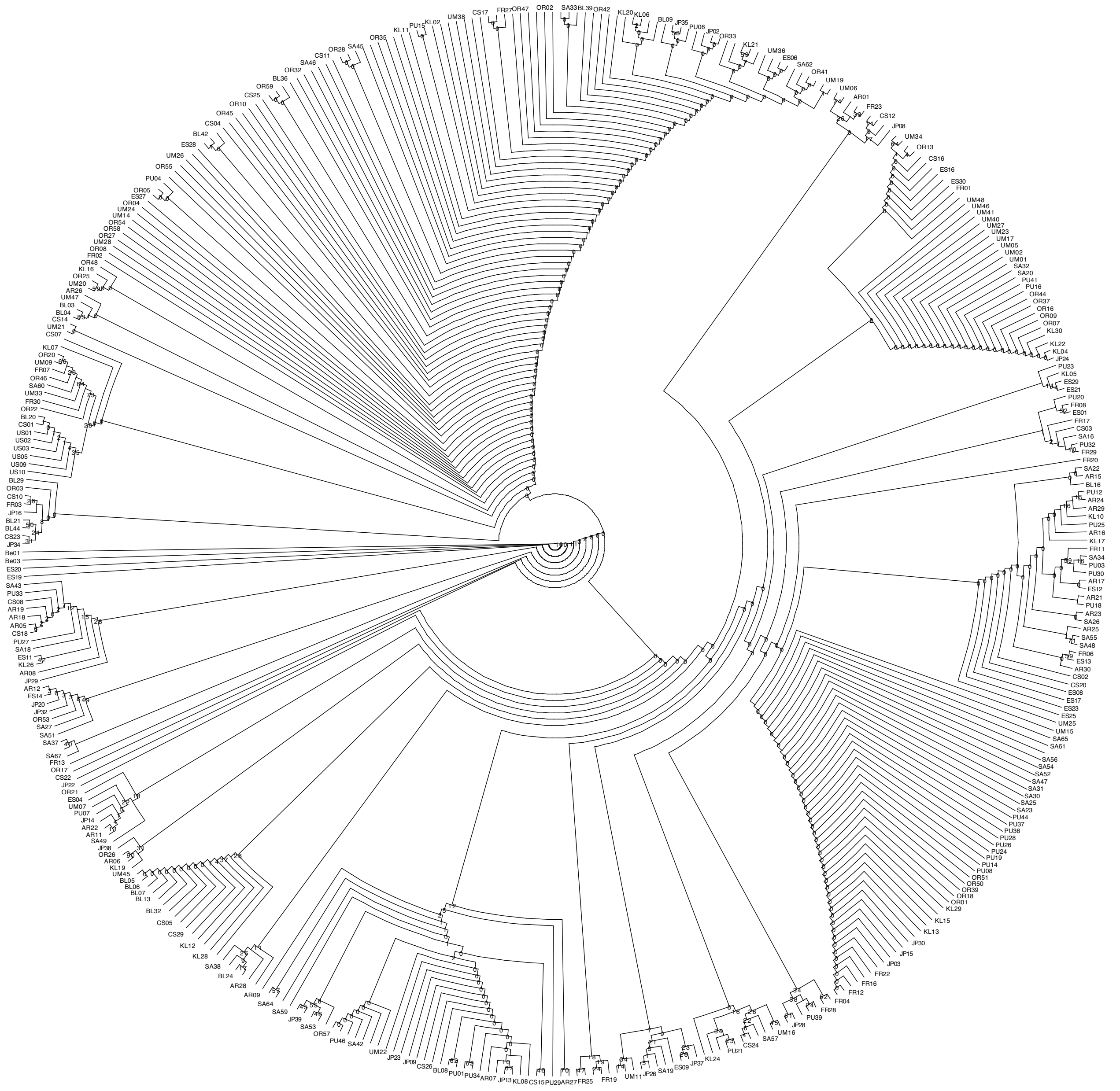

Supplement: S3 Fig — The robustness of branches was tested with 100 bootstraps, and a COI sequence of Balanus eburneus was used as an out-group for this maximum likelihood analysis. (PDF) [file pone.0147082.s003.pdf]
