# Supplementary material for: The Story of a Hitchhiker: Population Genetic Patterns in the Invasive Barnacle Balanus(Amphibalanus) improvisus Darwin 1854
Source: PLoS One. 2016 Jan 28;11(1):e0147082. doi: 10.1371/journal.pone.0147082 (PMC4731558; doi:10.1371/journal.pone.0147082)
Supplement: S3 Table — The populations are: Argentina (AR), France (FR), Saltö (SA), Kiel (KL), Torhamn (TO), Estonia (ES), Öregrund (OR), Umeå (UM), Black Sea (BL), Caspian Sea (CS), Japan (JP) and Pacific US (PU). (PDF) [file pone.0147082.s009.pdf]

S3 Table. Haplotype list with frequencies in all sampled populations of *B. improvisus*. Populations: Argentina (AR), France (FR), Saltö (SA), Kiel (KL), Torhamn (TO), Estonia (ES), Öregrund (OR), Umeå (UM), Black Sea (BL), Caspian Sea (CS), Japan (JP) and Pacific US (PU).

Haplotype/Population	PU	CB	NC	AR	FR	SA	KL	TO	ES	OR	UM	BL	CS	JP	N/hapl
	42	*7*	*7*	*30*	*29*	*49*	*30*	*29*	*30*	*57*	*50*	*26*	*30*	*32*	
BIH001	0.214	0	0	0.267	0.276	0.204	0.167	0.0345	0.133	0.0877	0.04	0.0385	0.0667	0.188	61
BIH002	0.167	0	0.714	0.0667	0.138	0.163	0.233	0.276	0.1	0.491	0.34	0.346	0.4	0.0625	112
BIH003	0.0476	0	0	0.0333	0	0.0408	0	0.0345	0.0333	0.0175	0.04	0.0769	0.0333	0.125	17
BIH004	0.0476	0	0	0	0.0345	0.0408	0.1	0.069	0.133	0.0877	0.2	0.0385	0.0333	0.0625	33
BIH005	0.0476	0	0	0	0	0	0	0	0	0	0	0	0	0	2
BIH006	0.0476	0	0	0	0	0	0	0	0	0	0	0	0	0	2
BIH007	0.0238	0	0	0.1	0	0.0204	0	0.0345	0	0	0	0	0.0333	0	7
BIH008	0.0238	0	0	0.0667	0	0	0.0333	0.0345	0	0	0	0	0	0	5
BIH009	0.0238	0	0	0.0667	0	0	0	0	0	0	0.02	0	0	0.0312	5
BIH010	0.0238	0	0	0	0	0	0.0333	0	0.0667	0	0	0	0	0	4
BIH011	0.0238	0	0	0	0	0	0	0	0	0	0.02	0	0	0	2
BIH012	0.0238	0	0	0	0	0	0	0	0	0	0	0	0	0.0312	2
BIH013	0.0238	0	0	0	0	0	0	0	0	0	0	0	0	0	1
BIH014	0.0238	0	0	0	0	0	0	0	0	0	0	0	0	0	1
BIH015	0.0238	0	0	0	0	0	0	0	0	0	0	0	0	0	1
BIH016	0.0238	0	0	0	0	0	0	0	0	0	0	0	0	0	1
BIH017	0.0238	0	0	0	0	0	0	0	0	0	0	0	0	0	1
BIH018	0.0238	0	0	0	0	0	0	0	0	0	0	0	0	0	1
BIH019	0.0238	0	0	0	0	0	0	0	0	0	0	0	0	0	1
BIH020	0.0238	0	0	0	0	0	0	0	0	0	0	0	0	0	1
BIH021	0.0238	0	0	0	0	0	0	0	0	0	0	0	0	0	1
BIH022	0.0238	0	0	0	0	0	0	0	0	0	0	0	0	0	1
BIH023	0.0238	0	0	0	0	0	0	0	0	0	0	0	0	0	1
BIH024	0.0238	0	0	0	0	0	0	0	0	0	0	0	0	0	1
BIH025	0	0.857	0.143	0	0	0	0	0	0	0	0	0.0385	0.0333	0	9
BIH026	0	0.143	0	0	0	0	0.0333	0	0	0	0	0.269	0.133	0	13
BIH027	0	0	0.143	0	0	0	0	0	0	0	0	0	0	0	1
BIH028	0	0	0	0.0667	0	0	0	0	0	0	0	0	0	0	2
BIH029	0	0	0	0.0333	0	0.0408	0	0	0.0333	0.0175	0	0	0	0.0625	7
BIH030	0	0	0	0.0333	0	0.0204	0.0333	0.0345	0	0	0	0	0.0333	0	5
BIH031	0	0	0	0.0333	0	0	0.0333	0	0	0	0	0	0	0	2
BIH032	0	0	0	0.0333	0	0	0.0333	0	0	0	0	0	0	0	2
BIH033	0	0	0	0.0333	0	0	0	0	0.0333	0	0.02	0	0	0	3
BIH034	0	0	0	0.0333	0	0	0	0	0	0	0	0	0	0	1
BIH035	0	0	0	0.0333	0	0	0	0	0	0	0	0	0	0	1
BIH036	0	0	0	0.0333	0	0	0	0	0	0	0	0	0	0	1
BIH037	0	0	0	0.0333	0	0	0	0	0	0	0	0	0	0	1
BIH038	0	0	0	0.0333	0	0	0	0	0	0	0	0	0	0	1
BIH039	0	0	0	0	0.0345	0	0.0333	0	0	0	0	0	0	0	2
BIH040	0	0	0	0	0.0345	0	0	0.0345	0	0.0175	0	0	0	0	3
BIH041	0	0	0	0	0.0345	0	0	0	0	0	0	0	0	0	1
BIH042	0	0	0	0	0.0345	0	0	0	0.0333	0	0	0	0	0	2
BIH043	0	0	0	0	0.0345	0	0	0	0.0333	0	0	0	0	0	2
BIH044	0	0	0	0	0.0345	0	0	0	0	0	0	0	0	0.0312	2
BIH045	0	0	0	0	0.0345	0	0	0	0	0	0	0	0	0	1
BIH046	0	0	0	0	0.0345	0	0	0	0	0	0	0	0	0	1
BIH047	0	0	0	0	0.0345	0	0	0	0	0	0	0	0	0	1
BIH048	0	0	0	0	0.0345	0	0	0	0	0	0	0	0	0	1
BIH049	0	0	0	0	0.0345	0	0	0	0	0	0	0	0	0	1
BIH050	0	0	0	0	0.0345	0	0	0	0	0	0	0	0	0	1
BIH051	0	0	0	0	0.0345	0	0	0	0	0	0	0	0	0	1
BIH052	0	0	0	0	0.0345	0	0	0	0	0	0	0	0	0	1
BIH053	0	0	0	0	0.0345	0	0	0	0	0	0	0	0	0	1
BIH054	0	0	0	0	0.0345	0	0	0	0	0	0	0	0	0	1
BIH055	0	0	0	0	0	0.0408	0	0	0	0	0	0	0	0	2
BIH056	0	0	0	0	0	0.0408	0	0	0	0	0	0	0	0	2
BIH057	0	0	0	0	0	0.0408	0	0	0	0	0	0	0	0	2
BIH058	0	0	0	0	0	0.0204	0.0333	0	0.0333	0	0.02	0	0	0.0312	5
BIH059	0	0	0	0	0	0.0204	0	0.0345	0	0.0175	0.02	0	0	0	4
BIH060	0	0	0	0	0	0.0204	0	0.0345	0	0	0	0	0	0	2
BIH061	0	0	0	0	0	0.0204	0	0	0	0.0175	0	0.0385	0	0	3
BIH062	0	0	0	0	0	0.0204	0	0	0	0	0	0	0	0	1
BIH063	0	0	0	0	0	0.0204	0	0	0	0	0	0	0	0	1
BIH064	0	0	0	0	0	0.0204	0	0	0	0	0	0	0	0	1
BIH065	0	0	0	0	0	0.0204	0	0	0	0	0	0	0	0	1
BIH066	0	0	0	0	0	0.0204	0	0	0	0	0	0	0	0	1
BIH067	0	0	0	0	0	0.0204	0	0	0	0	0	0	0	0	1
BIH068	0	0	0	0	0	0.0204	0	0	0	0	0	0	0	0	1
BIH069	0	0	0	0	0	0.0204	0	0	0	0	0	0	0	0	1

S3 Table continued. Haplotype list with frequencies in all sampled populations of *B. improvisus*.

Haplotype/Population	PU	CB	NC	AR	FR	SA	KL	TO	ES	OR	UM	BL	CS	JP	N/hapl
continued…	*42*	*7*	*7*	*30*	*29*	*49*	*30*	*29*	*30*	*57*	*50*	*26*	*30*	*32*	
BIH070	0	0	0	0	0	0.0204	0	0	0	0	0	0	0	0	1
BIH071	0	0	0	0	0	0.0204	0	0	0	0	0	0	0	0	1
BIH072	0	0	0	0	0	0.0204	0	0	0	0	0	0	0	0	1
BIH073	0	0	0	0	0	0.0204	0	0	0	0	0	0	0	0	1
BIH074	0	0	0	0	0	0.0204	0	0	0	0	0	0	0	0	1
BIH075	0	0	0	0	0	0	0.0333	0	0	0	0.02	0	0	0	2
BIH076	0	0	0	0	0	0	0.0333	0	0	0	0	0	0	0	1
BIH077	0	0	0	0	0	0	0.0333	0	0	0	0	0	0	0	1
BIH078	0	0	0	0	0	0	0.0333	0	0	0	0	0	0	0	1
BIH079	0	0	0	0	0	0	0.0333	0	0	0	0	0	0	0	1
BIH080	0	0	0	0	0	0	0.0333	0	0	0	0	0	0	0	1
BIH081	0	0	0	0	0	0	0.0333	0	0	0	0	0	0	0	1
BIH082	0	0	0	0	0	0	0	0.0345	0	0.0175	0	0	0	0	2
BIH083	0	0	0	0	0	0	0	0.0345	0	0.0175	0	0	0	0	2
BIH084	0	0	0	0	0	0	0	0.0345	0	0	0	0	0.0333	0	2
BIH085	0	0	0	0	0	0	0	0.0345	0	0	0	0	0.0333	0	2
BIH086	0	0	0	0	0	0	0	0.0345	0	0	0	0	0	0	1
BIH087	0	0	0	0	0	0	0	0.0345	0	0	0	0	0	0	1
BIH088	0	0	0	0	0	0	0	0.0345	0	0	0	0	0	0	1
BIH089	0	0	0	0	0	0	0	0.0345	0	0	0	0	0	0	1
BIH090	0	0	0	0	0	0	0	0.0345	0	0	0	0	0	0	1
BIH091	0	0	0	0	0	0	0	0.0345	0	0	0	0	0	0	1
BIH092	0	0	0	0	0	0	0	0.0345	0	0	0	0	0	0	1
BIH093	0	0	0	0	0	0	0	0	0.0333	0	0	0	0.0333	0.0312	3
BIH094	0	0	0	0	0	0	0	0	0	0	0.02	0	0	0	1
BIH095	0	0	0	0	0	0	0	0	0.0333	0.0175	0	0	0	0	2
BIH096	0	0	0	0	0	0	0	0	0.0333	0	0	0	0	0.0312	2
BIH097	0	0	0	0	0	0	0	0	0.0333	0	0	0	0	0	1
BIH098	0	0	0	0	0	0	0	0	0.0333	0	0	0	0	0	1
BIH099	0	0	0	0	0	0	0	0	0.0333	0	0	0	0	0	1
BIH100	0	0	0	0	0	0	0	0	0.0333	0	0	0	0	0	1
BIH101	0	0	0	0	0	0	0	0	0.0333	0	0	0	0	0	1
BIH102	0	0	0	0	0	0	0	0	0.0333	0	0	0	0	0	1
BIH103	0	0	0	0	0	0	0	0	0.0333	0	0	0	0	0	1
BIH104	0	0	0	0	0	0	0	0	0.0333	0	0	0	0	0	1
BIH105	0	0	0	0	0	0	0	0	0	0.0175	0.02	0	0	0	2
BIH106	0	0	0	0	0	0	0	0	0	0.0175	0.02	0	0	0	2
BIH107	0	0	0	0	0	0	0	0	0	0.0175	0	0	0	0.0312	2
BIH108	0	0	0	0	0	0	0	0	0	0.0175	0	0	0	0	1
BIH109	0	0	0	0	0	0	0	0	0	0.0175	0	0	0	0	1
BIH110	0	0	0	0	0	0	0	0	0	0.0175	0	0	0	0	1
BIH111	0	0	0	0	0	0	0	0	0	0.0175	0	0	0	0	1
BIH112	0	0	0	0	0	0	0	0	0	0.0175	0	0	0	0	1
BIH113	0	0	0	0	0	0	0	0	0	0.0175	0	0	0	0	1
BIH114	0	0	0	0	0	0	0	0	0	0.0175	0	0	0	0	1
BIH115	0	0	0	0	0	0	0	0	0	0.0175	0	0	0	0	1
BIH116	0	0	0	0	0	0	0	0	0	0	0.02	0	0	0	1
BIH117	0	0	0	0	0	0	0	0	0	0	0.02	0	0	0	1
BIH118	0	0	0	0	0	0	0	0	0	0	0.02	0	0	0	1
BIH119	0	0	0	0	0	0	0	0	0	0	0.02	0	0	0	1
BIH120	0	0	0	0	0	0	0	0	0	0	0.02	0	0	0	1
BIH121	0	0	0	0	0	0	0	0	0	0	0.02	0	0	0	1
BIH122	0	0	0	0	0	0	0	0	0	0	0.02	0	0	0	1
BIH123	0	0	0	0	0	0	0	0	0	0	0.02	0	0	0	1
BIH124	0	0	0	0	0	0	0	0	0	0	0.02	0	0	0	1
BIH125	0	0	0	0	0	0	0	0	0	0	0.02	0	0	0	1
BIH126	0	0	0	0	0	0	0	0	0	0	0	0.0769	0	0	2
BIH127	0	0	0	0	0	0	0	0	0	0	0	0.0769	0	0	2
BIH128	0	0	0	0	0	0	0	0	0	0	0	0	0.0333	0	1
BIH129	0	0	0	0	0	0	0	0	0	0	0	0	0.0333	0	1
BIH130	0	0	0	0	0	0	0	0	0	0	0	0	0.0333	0	1
BIH131	0	0	0	0	0	0	0	0	0	0	0	0	0.0333	0	1
BIH132	0	0	0	0	0	0	0	0	0	0	0	0	0	0.0625	2
BIH133	0	0	0	0	0	0	0	0	0	0	0	0	0	0.0312	1
BIH134	0	0	0	0	0	0	0	0	0	0	0	0	0	0.0312	1
BIH135	0	0	0	0	0	0	0	0	0	0	0	0	0	0.0312	1
BIH136	0	0	0	0	0	0	0	0	0	0	0	0	0	0.0312	1
BIH137	0	0	0	0	0	0	0	0	0	0	0	0	0	0.0312	1
BIH138	0	0	0	0	0	0	0	0	0	0	0	0	0	0.0312	1
BIH139	0	0	0	0	0	0	0	0	0	0	0	0	0	0.0312	1
